# First insights into the Drivers of the Cloacal Microbiome of the Wild Platypus (*Ornithorhynchus anatinus*)

**DOI:** 10.1007/s00248-026-02788-1

**Published:** 2026-05-12

**Authors:** Jana Stewart, Lucy E. Ockert, Tahneal Hawke, Michelle Power, Gilad Bino

**Affiliations:** 1 Centre for Ecosystem Science, School of Biological, Earth and Environmental Sciences, UNSW, Sydney, NSW 2052 Australia; 2 Evolution and Ecology Research Centre, School of Biological, Earth and Environmental Sciences, UNSW, Sydney, NSW 2052 Australia; 3 Taronga Institute of Science and Learning, Taronga Conservation Society Australia, Dubbo, NSW 2830 Australia; 4 School of Natural Sciences, Faculty of Science and Engineering, Macquarie University, North Ryde, Sydney, NSW 2009 Australia

**Keywords:** Reproductive biology, Conservation biology, Biogeography, Wildlife monitoring, Monotreme, Aquatic microbiome

## Abstract

The host microbiome plays a critical role in wildlife health, reproduction, and environmental responses. The platypus (*Ornithorhynchus anatinus*), a semi-aquatic monotreme endemic to eastern Australia, has remained microbiologically understudied despite conservation concern. Here, we present the first characterisation of the wild platypus cloacal microbiome using 16 S rRNA amplicon sequencing of samples collected across its eastern range, including sites recently affected by drought and bushfire. We found that region, and environmental disturbances such as bushfire and drought were significant drivers of bacterial community structure and composition, with influence from sex and breeding season also. Bushfire and drought both disrupted microbial community structure. The microbiome partially recovered following low-severity fires, but not after severe fire or prolonged drought, suggesting that microbiome resilience is linked to disturbance intensity. These findings suggest that the platypus microbiome is sensitive to environmental pressures and may offer a minimally invasive indicator of individual and ecosystem health. In addition to these ecologically important findings, across all sampled regions the platypus cloacal microbiome was consistently dominated by *Campylobacterota* and *Fusobacteriota*, with *Pseudomonadota* and *Bacillota* also prominent in the core microbiomes. Our results provide a critical baseline for integrating microbial health into conservation planning for this unique mammal.

**Supplementary Information:**

The online version contains supplementary material available at 10.1007/s00248-026-02788-1.

## Introduction

The platypus (*Ornithorhynchus anatinus*), an iconic semi-aquatic monotreme endemic to eastern Australia and Tasmania, occupies a mosaic of riverine habitats, from cool upland creeks to warmer lowland rivers [1]. Although a flagship species for freshwater conservation in Australia, relatively little is known about the platypus microbiome and its role in health and resilience [2]. The platypus is evolutionarily distinct due to its unique reproductive characteristics and evolutionary lineage that stretches back millions of years [3, 4]. Unlike therian mammals, platypuses have a cloaca, similar to reptile and avian species, connecting the gut microbiome to the reproductive microbiome [5]. Platypuses also possess a relatively simple digestive system, with their largely reduced stomach lacking gastric glands, relying instead on alternative, poorly understood mechanisms for digestion [6]. Additionally, monotremes have a lower body temperature than therian mammals or avian species (31–32 °C), which may facilitate a unique microbial community [7]. Cloacal microbiomes in other species have provided information on reproductive state and behaviour, and were also linked to offspring survival [8–10]. Given that digestion, metabolism, and health are tightly linked, characterising the platypus cloacal microbiome may illuminate the metabolic needs and physiological adaptations of this species.

Until recently, knowledge of the platypus microbiome was undocumented. The first characterisation of platypus gut bacterial communities was recently published, using faecal samples from captive (*n* = 17) and wild (*n* = 5) individuals from one location in the southern part of the platypus distribution [2]. Dungan & Thomas (2024) revealed a microbiome dominated by *Bacillota*, *Pseudomonadota*, *Fusobacteria*, and *Bacteroidota*, with 21 core bacterial taxa identified across individuals. Significant differences in community composition were observed between wild and captive platypuses, while alpha diversity (measured using observed ASVs, Shannon’s index, and inverse Simpson’s index) was not significantly different. Captive animals exhibited elevated levels of *Enterococcus*, a genus in the *Bacillota* phylum, associated with dysbiosis and potential pathogenicity, while wild individuals harboured greater abundances of *Bacillota* genera *Clostridium*, and *Epulopiscium*, *Cetobacterium* genus in the *Fusobacterota* phylum, and the *Pseudomonadota* genus *Rickettsiella* [2]. Despite the important findings from Dungan and Thomas (2024), broader understanding of the wild platypus microbiome across its geographical range is unknown.

Habitat disturbance can lead to a reduction in microbial diversity and an increase in opportunistic or pathogenic taxa, which may compromise host health [11]. Extreme climatic events, including fire and drought, can be particularly disruptive, modifying not only physical habitats but also the microbial reservoirs in soil and water that influence gut colonisation [12, 13], as well as the diversity and abundance of food sources such as invertebrates and plants [14, 15]. The impact of extreme fire events on wildlife microbiomes is relatively unknown with few studies having investigated this interaction [16, 17]. Understanding how disturbances such as bushfires and drought could potentially alter the platypus microbiome is important for predicting the species’ resilience, particularly as these events are common throughout the platypuses distribution [18].

To establish the platypus microbiome as a health indicator, this study aims to advance our understanding of key factors that shape the wild platypus cloacal microbiome, particularly the effects of biogeography, sex, and environmental disturbance such as fire. By comparing microbiomes across multiple river systems, we seek to identify how environmental and geographic variation shape microbial communities in wild platypus populations. As the cloaca combines gut and reproductive systems, we also compare potential differences between male and female cloacal microbiomes and the influence of breeding season. Finally, we assess the potential impact of environmental disturbance such as severe drought and bushfire on the platypus cloacal microbiome, and whether microbial communities recovered following the bushfire. Together, these analyses aim to provide new insights into the ecological drivers of microbial variation in wild platypuses and to inform conservation strategies that incorporate microbial health as an integral component of species resilience.

## Methods

### Sample Locations

**Fig. 1:**
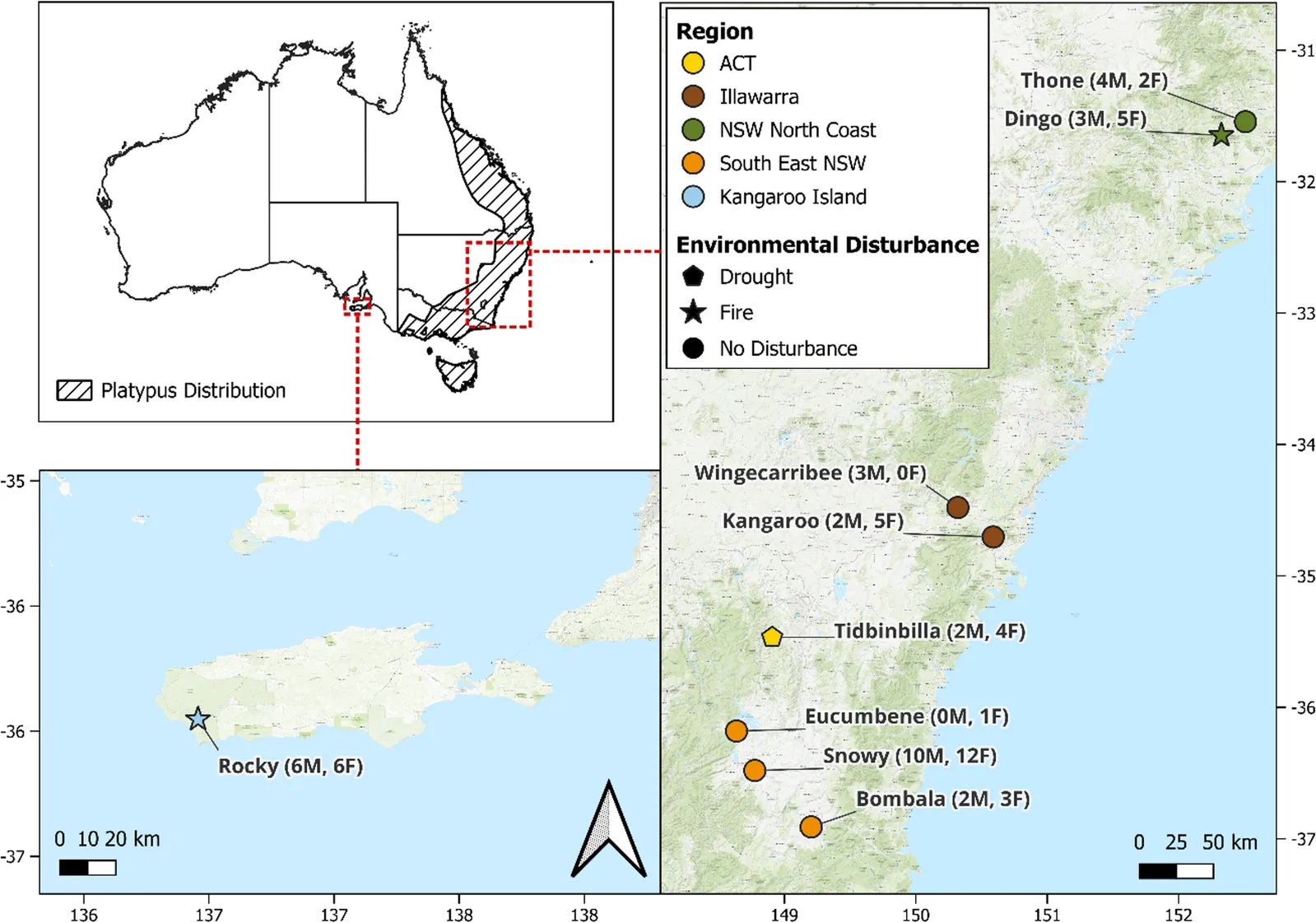
Map of sampling locations within the platypus distribution along the east coast of Australia and Kangaroo Island in South Australia (top left inset). Species distribution outlined on the inset map was sourced from Burbidge (2014) [20]. Samples from each river are divided by region denoted by colour. Starred markers indicate the presence of a fire at the site within the 18 months prior to sampling. Pentagon marker denotes severe drought. Numbers in brackets denote the sample size of (M) male and (F) female platypus for that river. Visualised using QGIS (v 3.30.2)

All platypus trapping and sampling was performed by trained and highly experienced researchers under the UNSW AEC ethics (19/142A and 22/130B), DPIE scientific licenses (SL101655 and LT201933), DPIE scientific collection permit (P15/0096 − 2.0 & OUT20/15426). In total 70 platypuses were sampled from five distinct regions across their distributional range: Australian Capital Territory (ACT), Illawarra (New South Wales; NSW), NSW North Coast, South East NSW, and Kangaroo Island (South Australia; SA). Within these regions, nine river systems were targeted, encompassing major catchments and representative habitat types, including drought affected, fire-affected and unburnt areas (Fig. 1; Table 1). Kangaroo Island was the only region with juveniles captured. Sub-adults were captured in Kangaroo Island (*n* = 1), Illawarra (*n* = 3), and South East NSW (*n* = 3) [19]. To account for potential contamination during field sampling and handling, air control samples were collected during each trapping period at every location, resulting in a total of 22 negative controls.

**Table 1:** Location and number of samples collected, stratified by sex, age, and fire impact

Region	River	Sex	Age	Fire	Count
ACT	Tidbinbilla	F	A	No	4
ACT	Tidbinbilla	M	A	No	2
Illawarra	Kangaroo	F	A	No	5
Illawarra	Kangaroo	M	A	No	2
Illawarra	Wingecarribee	M	SA	No	3
Kangaroo Island	Rocky	F	A	Yes	4
Kangaroo Island	Rocky	F	J	Yes	2
Kangaroo Island	Rocky	M	A	Yes	2
Kangaroo Island	Rocky	M	J	Yes	3
Kangaroo Island	Rocky	M	SA	Yes	1
NSW North Coast	Dingo	F	A	Yes	5
NSW North Coast	Dingo	M	A	Yes	3
NSW North Coast	Thone	F	A	No	2
NSW North Coast	Thone	M	A	No	4
South East NSW	Bombala	F	A	No	3
South East NSW	Bombala	M	A	No	1
South East NSW	Bombala	M	SA	No	1
South East NSW	Eucumbene	F	A	No	1
South East NSW	Snowy	F	A	No	12
South East NSW	Snowy	M	A	No	8
South East NSW	Snowy	M	SA	No	2

### Sample Collection

We live trapped platypuses between November 2018 and January 2022 using two primary methods: unweighted mesh nets and fyke nets. Sample times varied with 46 collected during breeding season when platypuses are most active, and 24 collected outside of breeding season. We deployed mesh nets (80 mm multifilament nets, 25 m × 2 m) using a 6-foot punt and set lengthwise across pools from approximately 16:00 until midnight, visually monitored the nets at regular intervals using a spotlight and immediately retrieved any captured individuals. We deployed fyke nets (30 mm knotless 20-ply nylon, 1 m × 5 m wings, 0.8 m × 5 m wings) in upstream and downstream pairs in shallow sections of suitable streams and checked every three hours overnight.

We anaesthetised captured individuals using isoflurane (Pharmachem; 2–5%) delivered in oxygen (2–3 L/min) following established protocols [21]. We weighed, measured (body, tail, and bill), and assessed each animal for sex and age based on spur morphology [22]. To ensure that we did not resample individuals, trapped platypuses were scanned for existing microchips and, if unmarked, implanted with a passive integrated transponder (PIT) tag (Trovan) for identification.

We collected cloacal microbiome samples by inserting sterile cotton swabs approximately 1 cm into the cloaca. For each field trip, we also collected a negative control air sample by waving a sterile swab in the air for 10 s. We preserved all swabs in 99.9% ethanol to maintain DNA integrity. Swabs were stored at − 80 °C within a week of field collection until DNA extraction.

### DNA Extraction and Sequencing

We performed all DNA extractions in a physical containment level two (PC2) laboratory at Macquarie University, Sydney, Australia. We extracted genomic DNA from platypus cloacal and control air swabs using the ZymoBIOMICS™ DNA Miniprep Kit (Zymo Research Corp., California, USA), following the manufacturer’s protocol. We then assessed DNA quantity and purity was using a NanoDrop One^C^ Microvolume UV-Vis Spectrophotometer (Thermo Scientific, Waltham, MA, USA). We sent extracted DNA to the Ramaciotti Centre for Genomics (UNSW, Sydney, Australia) for 16S amplicon sequencing following the Earth Microbiome Project (EMP) protocol [23]. Briefly, Ramaciotti prepared 16S rRNA gene libraries targeting the V4 hypervariable region using the 515F (GTGYCAGCMGCCGCGGTAA-3’) [24] and 806R (GGACTACNVGGGTWTCTAAT-3’) [25] primer pair. They quantified and normalised DNA extracts prior to polymerase chain reaction (PCR) amplification and combined equimolar amounts of each amplicon to generate a pooled library. They sequenced the pooled library on an Illumina MiSeq platform using paired-end 2 × 250 bp chemistry and included a PhiX control spike-in as an internal sequencing quality standard. A yield of 8–12 million read pairs per run was targeted, with quality control thresholds requiring > 75% of bases to exceed Q30.

### Bioinformatics and Statistical Analysis

We filtered and trimmed raw sequencing reads in R (v 4.3.3) using the *DADA2* (v 1.28.0) package [26]. We trimmed bases with a quality score of less than 30 (< Q30), truncated forward and reverse reads at 240 bp and 200 bp, respectively, and then merged the forward and reverse reads based on default parameters (minimum overlap = 12 bp, maximum mismatches = 0). We removed chimeric sequences using the consensus method as part of the ‘removeBimeraDenovo*’* function in *DADA2*. To assign taxonomy to the species level, we constructed an amplicon sequence variant (ASV) table and trained the classifier on the SILVA 138.2 dataset [27, 28] formatted for *DADA2.* Species level taxonomy was assigned based on exact (100%) matching between ASVs and sequenced reference strains, as this is recognised as the most appropriate way to assign species to 16 S gene fragments [29]. To remove contaminating ASVs from the cloacal samples, we used the ‘prevalence’ method in *decontam* (v 1.20.0) [30], which utilises a chi-square test to generate the likelihood of each sequence feature being a contaminant, represented by a prevalence score (*p*-value). We identified ASVs as contaminants when they were present at a higher prevalence in the air control samples than in the cloacal samples (prevalence score threshold = 0.5) and applied the ‘batch’ argument where each batch was classified as a singular field trip. To retain only bacterial ASVs for downstream analyses, we also removed any ASVs assigned to mitochondria, chloroplasts, or archaea, as well as those lacking a kingdom-level taxonomic assignment (i.e. non-target ASVs).

To visualise the relative abundance of bacterial taxa at phylum, family, and genus levels in each sample, we used *phyloseq* (v1.54.1) [31] to agglomerate counts at each taxonomic rank and converted counts to relative abundance (%). We grouped taxa with a mean relative abundance < 0.25% into ‘Other’ and generated stacked bar plots for each sample using *ggplot2* (v 4.0.2) [32], grouping samples by geographic region and ordering by sex.

To examine microbial alpha diversity, we rarefied the dataset to 21,144 reads per sample to balance read retention with sample retention, given the wide range of library sizes across samples. The rarefaction depth of 21,144 reads was selected based on rarefaction curves and sample read depths (Supplementary Table S1, Figure S1). Samples below this threshold had substantially lower read depths, and reducing the threshold would therefore retain few additional samples while considerably reducing the rarefied read count. Excluded samples spanned multiple locations and both sexes and were therefore not biased towards any location or sex. Samples below the rarefaction threshold were retained for compositional analyses using alternative normalisation methods and were therefore only excluded from alpha diversity analyses. This led to 12 samples being dropped due to low read counts, including individuals from ACT (*n* = 1 M), NSW North Coast (*n* = 3 F, 2 M), Kangaroo Island (*n* = 1 F, 2 M), Illawarra (*n*= 1 F, 1 F), and South East NSW (*n* = 1 F). We then assessed alpha diversity using the ‘alpha’ function in the *microbiome* package (v 1.32.0) [33] to calculate the number of observed ASVs, Shannon’s Diversity Index, Simpson Evenness Index, and Dominance Index. An Analysis of Variance (ANOVA) was performed for each alpha diversity metric to test for statistical differences between sex and region. Both the ANOVA and beta dispersion homogeneity test (‘betadisper’) were performed using the ‘vegan’ package (v 2.7-3) [34].

To examine how sex and region shape the cloacal microbial community structure in platypuses, we normalised our data using a total sum scaling (TSS) transformation which allowed us to retain all samples. We then used a Bray-Curtis similarity matrix and non-metric multidimensional scaling (nMDS) to visually compare community structure. To examine whether region or sex had a significant influence on the community structure, we used a differential abundance analysis with the ‘manyglm’ function in the *mvabund* package (v 4.2.1) [35]. This model-based approach simultaneously fits a multivariate generalised linear model (GLM) to all ASVs present to determine community-level responses to variables of interest, then uses likelihood ratios to identify which ASVs are driving the response. It is well suited to highly variable microbial communities, and by modelling abundance data directly and accounting for mean–variance relationships, provides robust inference without relying on data transformations, normalisations or distance-based methods [36, 37]. We then determine significant community-level responses using ‘anova.manyglm’ with an adjusted univariate *p*-value to account for multiple testing. Due to limited sample sizes within each region and breeding season, only additive effects of region, sex, and season could be modelled.

To better understand if taxa present in the microbial community were functionally important across all regions, we examined the core microbiome using the ‘core_members’ function from the *microbiome* package. The core microbiome identifies taxa that are ubiquitous in a community by assessing occurrence and abundance thresholds, providing insight into which taxa are functionally important. We determined the core microbiome to be ASVs which were prevalent in at least 70% of the samples within a region at 0.001% abundance. The prevalence threshold aims to balance the detection of widespread taxa while accounting for natural variation among hosts, while the abundance threshold aims to include all taxa consistently present, including low-abundance ASVs that may contribute to host or ecosystem function [38, 39]. We selected our prevalence threshold based on recent literature which suggested a threshold of ASV occurrence ranging between 50–100% [40]. As our samples came from a wide range of habitats with some populations having undergone environmental stress, we expected there may be some taxa lost in some of these disturbed populations. Our aim for establishing a core microbiome was to identify any taxa consistent across all populations which could provide a foundation for key functional taxa. Once core ASVs were identified, taxonomy was then agglomerated at Genus level.

To determine the impact of environmental disturbances (i.e. bushfire) on the platypus cloacal microbiome, we compared bacterial community structure using a multivariate linear model (‘manylm’ in *mvabund*). We compared individuals from rivers that had experienced recent bushfire (Dingo: NSW North Coast, 6 months and 18 months post-fire; Rocky: Kangaroo Island, 16 months post-fire) with either nearby rivers (1.9 km away) which had not experienced fire (Thone: NSW North Coast) or with individuals from the same river at a later time point to assess recovery (Rocky: Kangaroo Island, 26 months post-fire). Due to the small sample size of these locations (*n* < 15 at each river), we were unable to separate samples by sex. To identify bacterial families which were most affected by fire, we ranked the top 10 families with the highest effect coefficient from the ‘manylm’ results for each region.

## Results

DNA extraction of cloacal swabs yielded concentrations ranging from 0.4 to 7.8 ng/µL. PCR amplification of the 16 S rRNA V4 region was successful for all samples, and all libraries yielded sequencing reads. A total of 92 samples were sequenced, consisting of 70 unique platypus cloacal swabs and 22 negative controls. The sequencing of the biological samples resulted in a total of 8,060,720 raw reads with a mean (± SD) of 115,153 ± 55,740 reads per sample, while the sequencing of the negative controls resulted in a total of 455,815 raw reads with a mean (± SD) of 20,719 ± 39,664 reads per control. In total, 10,174 unique ASVs were initially generated. One environmental control from Rocky, Kangaroo Island (KI-14) was excluded from further analysis due to its elevated read count and the collection of an additional negative control during this trip which could be used for the identification and removal of contaminating ASVs. *decontam* identified 53 ASVs as contaminants, which were subsequently removed from the dataset prior to downstream analysis (Figure S1, Table S2). After filtering, trimming, decontamination, and the removal of non-targeted taxa and 3405 chimeras, 5,025 unique ASVs remained. The final dataset consisted of 3,965,495 reads across 70 cloacal samples, with a mean (± SD) of 56,650 ± 34,337 processed reads per sample. Taxonomic assignment resulted in 37 unique phyla, 348 unique families, 816 unique genera, and 274 unique species, though many at species level were unclassified.

### Sex Differences in Microbial Community Structure and Composition

**Fig. 2:**
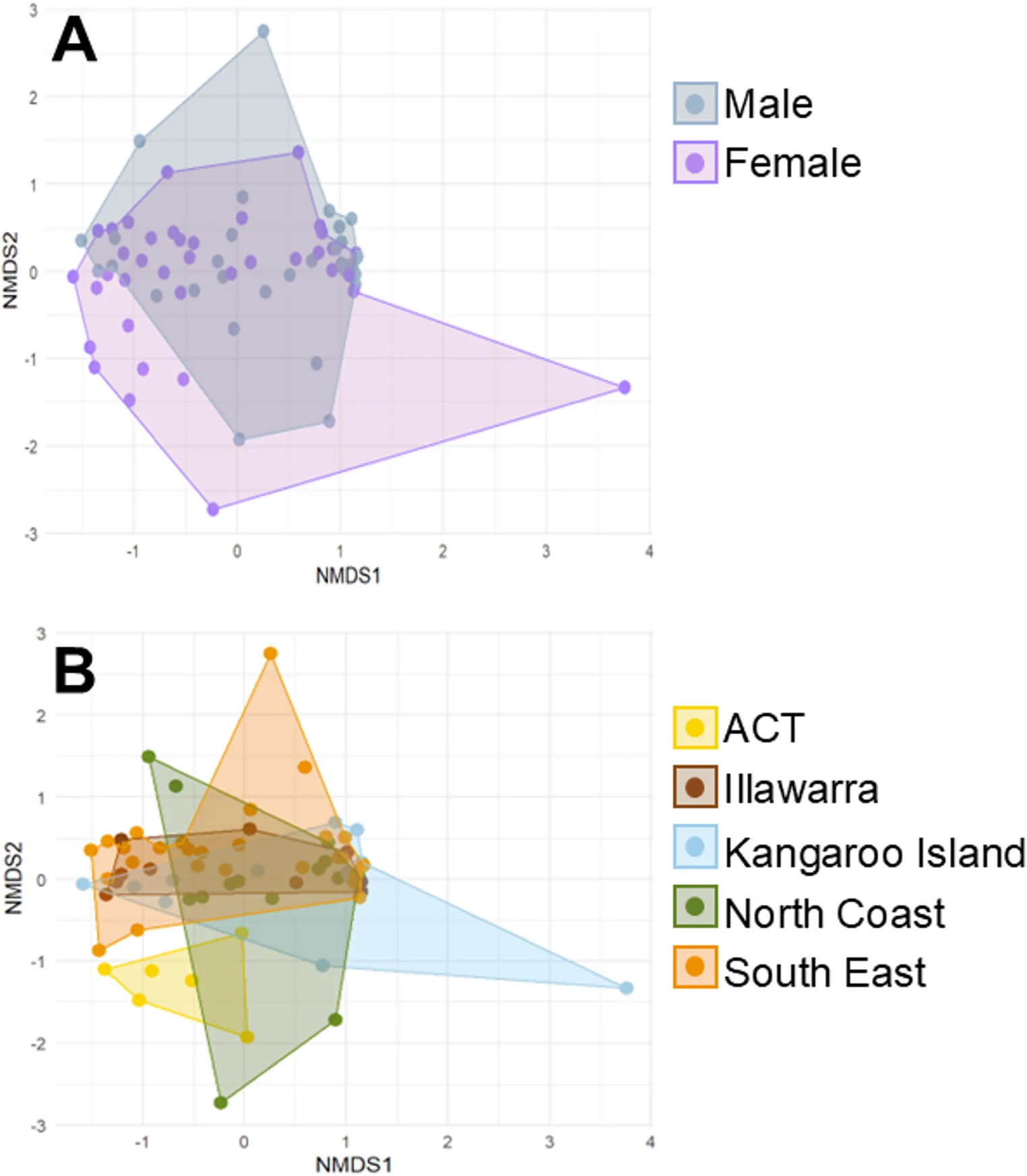
nMDS plots comparing the bacterial community structure between (**A**) males (*n* = 42) and females (*n* = 38), and (**B**) regions (ACT, *n* = 6; Illawarra, *n* = 10; Kangaroo Island, *n* = 12; NSW North Coast, *n* = 14; South East NSW, *n* = 38)

Using rarefied samples, sex was not a significant factor shaping cloacal microbiome alpha diversity; observed richness, Shannon diversity, Simpson evenness, and dominance were all non-significant (*p* > 0.3), and beta dispersion did not significantly differ between sexes (F = 0.0023, *p* = 0.9615). Using TSS normalised samples, nMDS based on Bray-Curtis similarity revealed some separation between sexes (Fig. 2A). The multivariate GLM (‘manyglm’) identified significant differences in microbial community composition between males and females (*p* < 0.005), though variation in sample read depths should be considered when interpreting this result.

**Fig. 3:**
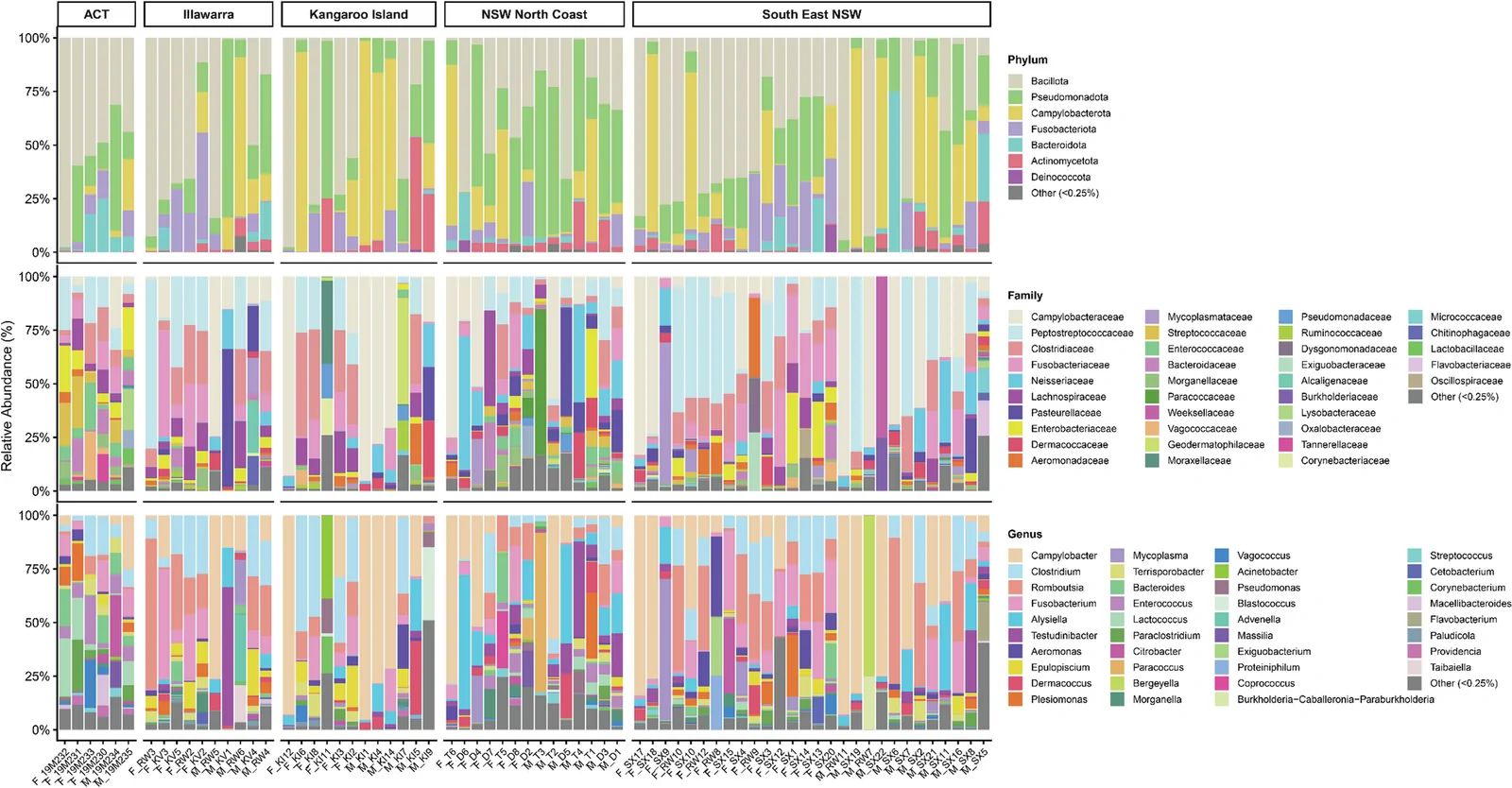
Relative abundance of bacterial taxa at phylum, family, and genus levels. Within each region, samples are ordered by sex, with F indicating females and M indicating males (ACT: F = 4, M = 2; Illawarra: F = 5, M = 5; Kangaroo Island: F = 6, M = 6; NSW North Coast: F = 7, M = 7; South East NSW: F = 16, M = 12). Taxa are ranked from highest to lowest mean relative abundance across all samples and the order of the legend (most abundant at the top). Taxa with a mean relative abundance below 0.25% are grouped into other

At the phylum level, sex-specific patterns in relative abundance were evident across most regions. Males generally exhibited higher proportions of *Campylobacterota*, while females harboured greater relative abundance of *Fusobacteriota* (Fig. 3). This pattern was consistent across all regions except the NSW North Coast. A model comparison between the additive effects of sex and region and a full interaction model revealed that the additive model provided a significantly better fit (*p* < 0.001), while the interaction model was only marginally non-significant (*p* = 0.059), suggesting independent but strong effects of both variables.

### Seasonal Variation in Microbial Community Structure and Composition

Breeding season also significantly influenced the cloacal microbiome composition, with significant differences in community structure between samples taken during breeding and non-breeding seasons (‘manyglm’; *p* = 0.022). However, no significant differences were found in any alpha diversity indices (Shannon diversity, Simpson evenness, observed ASVs, or dominance) after rarefying. Only three of the five regions had samples from both breeding and non-breeding seasons and this should be considered with these results (Table 1). Due to limited sample sizes and the lower effect of season on the cloacal microbiome, we did not include it in further downstream analyses and rather focused on sex and region.

### Regional Variation in Microbial Community Structure and Composition

Microbial community structure varied by region (Fig. 2B), with each region exhibiting a distinct bacterial community (‘manyglm’; *p* < 0.005). Using the rarefied dataset, observed richness (*p* = 0.038, F = 2.749, R^2^ = 0.172) and Simpson diversity (*p* = 0.003, F = 4.675, R^2^ = 0.261) were significantly different between regions, though differences in Simpson evenness and dominance across regions were not statistically significant. Beta dispersion was not significant (F = 0.732, *p* = 0.5734). Overall, phylum-level composition varied among regions, with *Campylobacterota*,* Bacillota*,* Pseudomonadota*, and *Fusobacteriota* dominant across most regions.

### Core Microbiome Composition

**Fig. 4:**
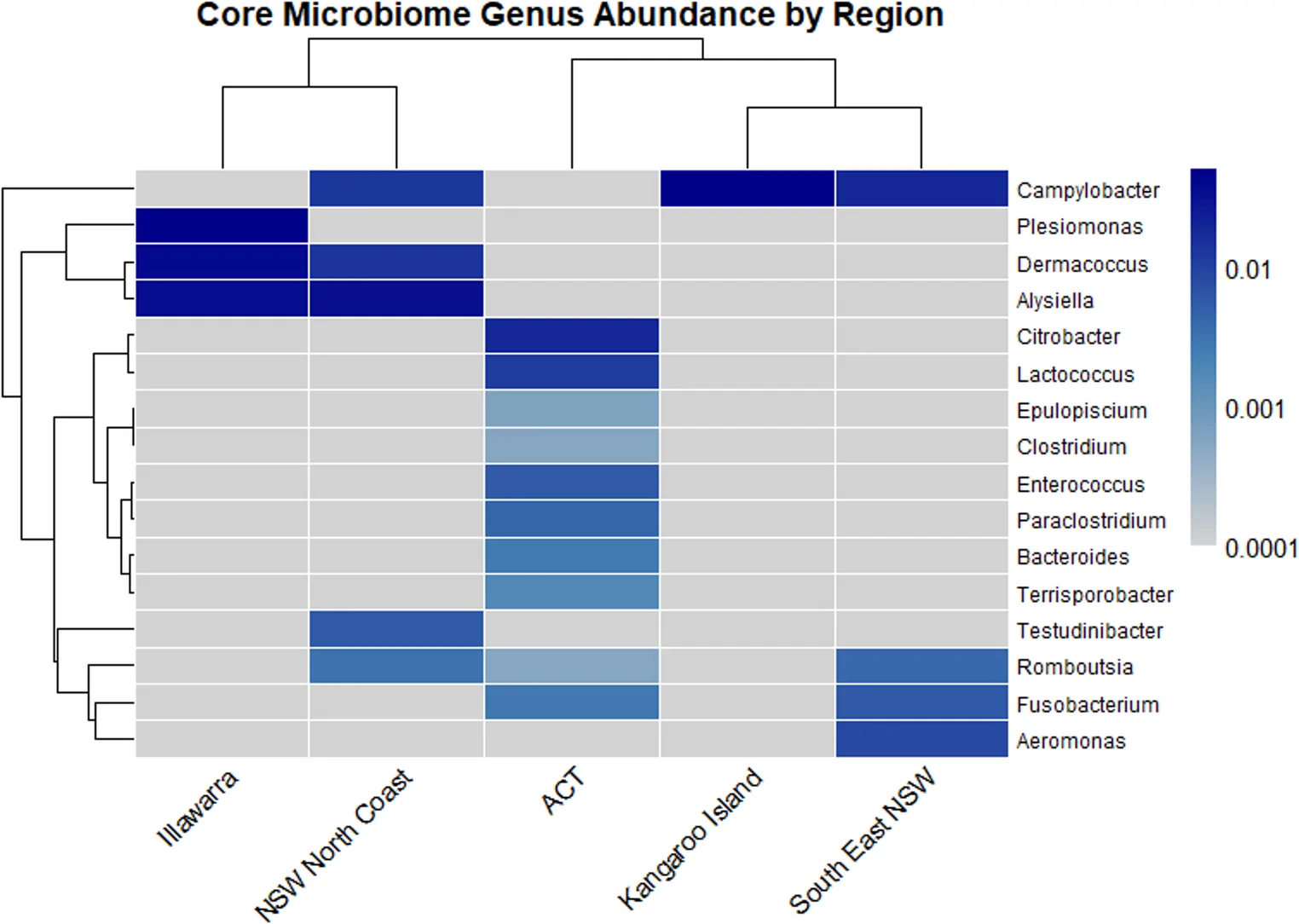
Clustered heatmap with dendrogram illustrating the bacterial genera making up the core microbiome of each region, with abundance log-scale transformed. The core microbiome was defined as bacterial genera present in ≥ 70% of samples at a minimum relative abundance of 0.001% in each region

Across all regions, 16 bacterial genera from six phyla were identified as members of the core microbiome. None of the genera were shared across all regions (Fig. 4). ACT had the most dissimilar core microbiome, while Illawarra and NSW North Coast were the most similar in composition and abundance, and were more similar to Kangaroo Island and South East NSW than to ACT (Fig. 4).

Core microbiome richness varied among regions. The ACT harboured the most diverse core microbiome, with 10 genera identified (*Lactococcus*,* Enterococcus*,* Paraclostridium*,* Terrisporobacter*,* Epulopiscium*,* Romboutsia*,* Clostridium*,* Bacteroides*,* Fusobacterium*,* Citrobacter*,* Dermacoccus*), eight of which were unique to this region. The Illawarra and NSW North Coast regions hosted three (*Dermacoccus*,* Plesiomonas*,* Alysiella*) and five (*Dermacoccus*,* Testudinibacter*,* Romboutsia*,* Campylobacter*,* Alysiella*) core genera respectively. South East NSW hosted four genera (*Romboutsia*,* Campylobacter*,* Fusobacterium*,* Aeromonas*) and Kangaroo Island hosted only one (*Campylobacter*).

### Impact of Bushfire and Drought Disturbance on the Cloacal Microbiome

**Fig. 5:**
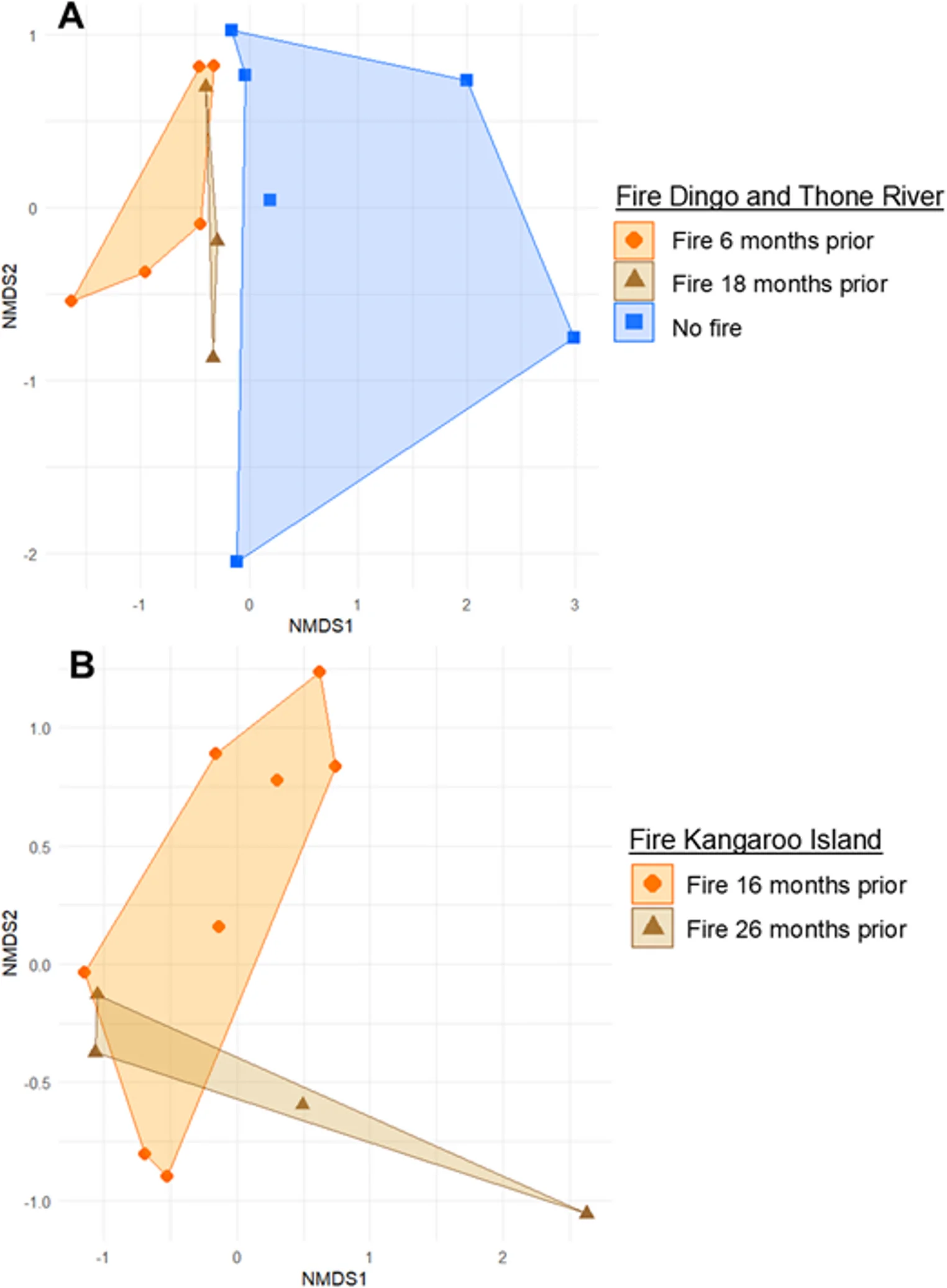
nMDS plots based on Bray-Curtis similarity comparing bacterial community structure in platypuses from fire-affected rivers. (**A**) Dingo river, sampled 6 and 18 months post-fire, compared with Thone River, a neighbouring unburnt river; (**B**) Kangaroo Island, sampled 16 and 26 months post-fire

Bushfire exposure was associated with significant alterations to the platypus cloacal microbiome, though the magnitude and persistence of these effects varied by region. In the NSW North Coast, community composition differed significantly between platypuses sampled six months post-fire in the Dingo River to those from the nearby unburnt Thone River (‘manyglm’; *p* = 0.005, F = 131.8). However, no significant difference was detected between the unburnt Thone River and samples taken 18 months post-fire in the Dingo River (*p* = 0.657), nor between the 6- and 18-month post-fire samples from the Dingo River (*p* = 0.816), although sample sizes were small and may impact these results. These patterns were reflected in nMDS ordinations (Fig. 5A), where 6-month post-fire samples clustered separately from both the unburnt and 18-month samples. On Kangaroo Island, bacterial community structure showed a trend toward difference between 16- and 26-month timepoints post-fire (‘manyglm’; *p* = 0.061, F = 76.79), but clustering on nMDS plots appeared less distinct (Fig. 5B).

**Fig. 6:**
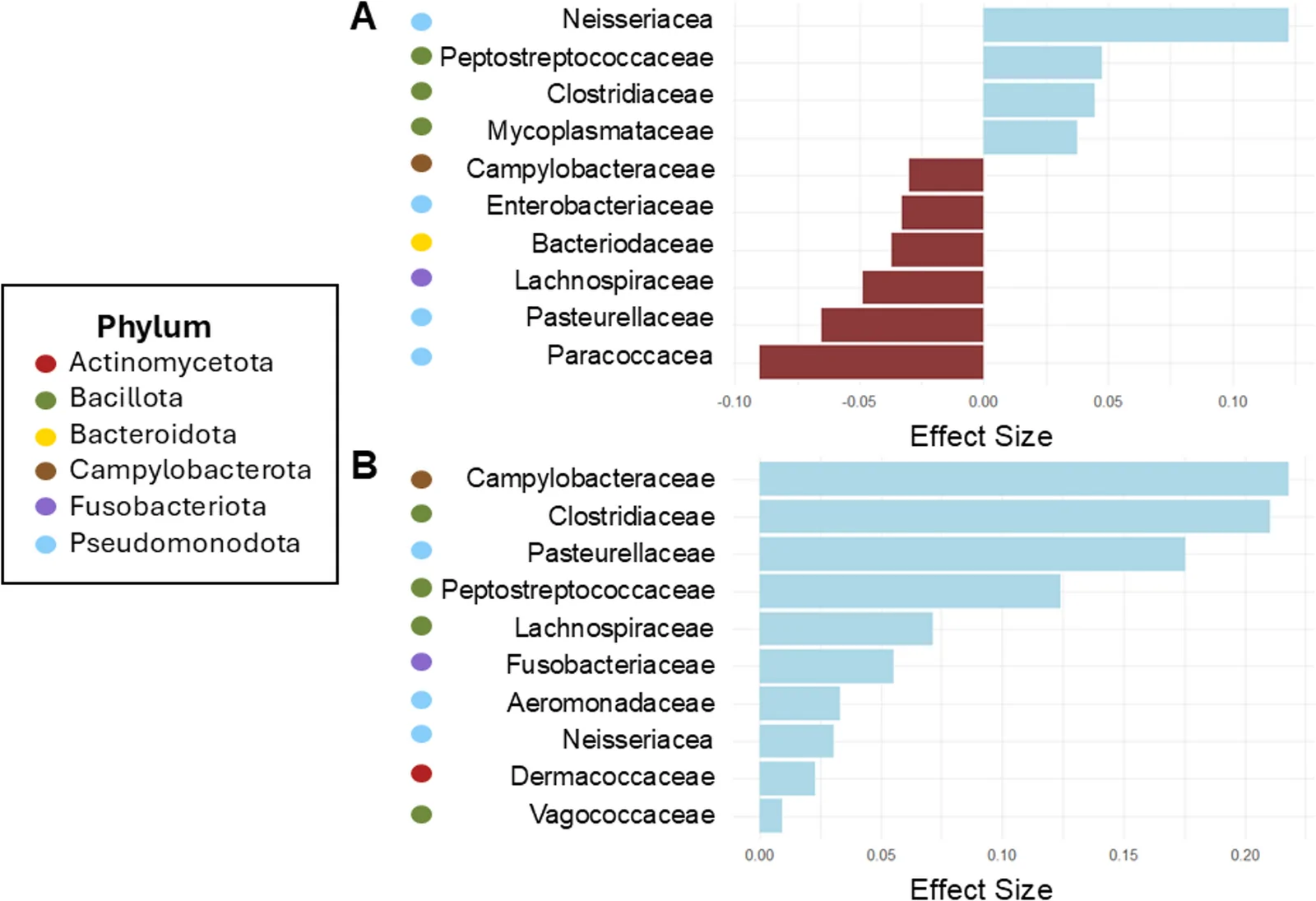
Bacterial families which were most affected by fire in (**A**) NSW North Coast 6 months post-fire compared to unburnt, and (**B**) Kangaroo Island 16 months post-fire compared to 26 months post-fire. Families are colour coded by phylum and effect direction is indicated by colour, positive (blue) and negative (maroon)

Our multivariate LM analysis did not identify any bacterial families significantly affected by bushfire, though this may be due to the small and uneven sample sizes. Although no individual bacterial families were significant after correction for multiple comparisons, the overall community-level tests justified exploration of taxon-level contributions. We therefore report the effect coefficients for the ten bacterial families showing the greatest magnitude of response to fire in each region as candidate taxa for future investigation. Using the effect coefficient, we identified the top 10 bacterial families most affected by fire in each region. In the NSW North Coast, four families increased and six decreased in abundance between fire-affected and unburnt rivers (Fig. 6A). On Kangaroo Island, all ten most-affected families increased in relative abundance from 16 to 26 months post-fire (Fig. 6B). While there was some overlap in affected taxa between regions, most fire-responsive families were region-specific.

The cloacal microbiome of ACT platypuses, which has experienced extreme drought, was significantly different from all other regions (*p* < 0.001). At the phylum level, ACT platypuses had higher relative abundance of *Bacillota* and *Bacteroidota* (Fig. 3). The ACT core microbiome also showed the greatest richness across regions (Fig. 4) and was predominantly composed of *Bacillota*, differing from the *Pseudomonadota* and *Campylobacterota* dominated core microbiome of the other regions (Fig. 4).

## Discussion

This study presents the first comprehensive characterisation of the wild platypus cloacal microbiome across multiple regions of its native range, as well as assessing the impact of environmental disturbances. The cloacal microbiome was dominated by *Campylobacterota* and *Fusobacteriota*, *Bacillota*, and *Pseudomonadota* which are commonly found in therian mammal microbiome communities [2, 41]. Our findings demonstrate that the platypus cloacal microbiome is influenced by environmental disturbance and region, suggesting plasticity in response to habitat conditions.

### Sex-based Differences and Breeding Season

Sex was inconsistent as a driver of cloacal microbiome composition in wild platypuses which may be linked to the differences in breeding season across samples. Our results found seasonal shifts in the cloacal microbiome, though due to sample sizes we were unable to fully explore this and clear conclusions cannot be made from these results. Sex-related microbial trends have been observed in other mammals, birds, and reptiles, which have been attributed to variation in hormone levels, behaviour, and reproductive physiology [9, 10, 42, 43]. Overall, females had a higher relative abundance of *Bacillota* (Female: mean = 49.39%, SE = 4.79%; Male: mean = 26.81%, SE = 5.33%) representing a 22.58% difference between sexes, and *Fusobacterota* (Female: mean = 10.83%, SE = 2.00%; Male: mean = 3.96%, SE = 1.18%) representing a 6.87% difference between sexes. *Campylobacterota* (Female: mean = 15.15%, SE = 4.20%; Male: mean = 27.39%, SE = 5.77%) and *Pseudomonadota* (Female: mean = 17.71%, SE = 2.86%; Male: mean = 28.66%, SE = 4.23%), however, were more 12.24% and 10.05% more abundant in male platypus respectively. Platypus activity varies with breeding season, with males travelling further with increased burrow visitation, and scent-marking [22, 44, 45], and females travelling further just after lactation at the end of the breeding season [22]. Platypus blood chemistry also shows seasonal variation [46], likely reflecting changes in metabolic activity. The observed compositional differences could reflect these behavioural and physiological processes, though further research is required.

### Regional Variation and Environmental Disturbance

Microbial community composition, species richness, and diversity varied significantly among regions. This pattern of regional differentiation is consistent with findings from other vertebrate species, where site-specific microbial communities are shaped by habitat conditions, diet, and local environmental microbiota [47, 48]. In platypuses, these differences may reflect variation in aquatic microbial reservoirs, prey composition, and catchment-level environmental quality across sites.

In the fire affected regions of NSW North Coast and Kangaroo Island, the increase in the bacterial families *Peptostreptococcaceae* and *Clostridiaceae* is consistent with other habitat disturbance studies in marsupials [49]. Microbiome composition initially differed from unburnt rivers but converged over time, indicating partial recovery. On Kangaroo Island, microbiome shifts were more subtle, potentially reflecting delayed recovery from compounded disturbances including drought, fire, and flooding [21]. These events drove persistent sedimentation, river degradation, and altered aquatic communities [21, 50], which likely influenced cloacal microbiota indirectly through changes in foraging behaviour, evidenced by increased crayfish consumption and reduced dietary diversity post-disturbance [51, 52]. Notably, the increased dominance of particular bacterial families in the cloacal microbiome during later sampling periods could reflect a narrower dietary profile or shifts in gut function associated with prey type [53, 54].

Severe drought had the most apparent effect on the cloacal microbiome composition which could be seen even at the phylum level and in the core microbiome, with increased abundance in Bacillota families, particularly *Streptococcaceae* which has also been associated with habitat disturbance in coyotes [55]. Platypuses from the drought affected ACT region were sampled as part of a translocation effort as the river had been greatly reduced to a handful of remnant pools [56]. Water quality deteriorates under drought [57], affecting macroinvertebrates and microbial communities [58, 59]. The combined effect of habitat quality reduction, reduced shelter, and shifts in diet availability would likely have a strong impact on platypus health and this is reflected in the cloacal microbiome. Taken together, these findings suggest that microbiome profiling may serve as a sensitive indicator of ecosystem disturbance.

### Core Microbiome Characteristics and Taxonomic Composition

Site specific core microbiomes could be seen particularly reflected in the ACT and Kangaroo Island regions which had undergone disturbance from drought and bushfire. The site specific associations seen even at the genus level, suggest that the cloacal microbiome can offer valuable information on animal health and environmental stress. The ACT platypus were most distinct and these genera may be useful as indicator taxa of stress.

Across all sampled regions, the platypus cloacal microbiome was consistently dominated by *Campylobacterota* and *Fusobacteriota*, with *Pseudomonadota* and *Bacillota* also prominent in the core microbiomes. This is similar to the faecal microbiota of captive and wild platypuses, which were typically dominated by *Bacillota* and *Pseudomonadota* [2]. The gut microbiota of most therian mammals are typically dominated by *Pseudomonadota* and *Bacillota* and are shaped by fermentative digestion and host diet [60]. The predominance of *Campylobacteraceae* and *Fusobacteriaceae* may reflect both the semi-aquatic ecology of platypuses and the anatomical and physiological properties of the cloaca. These families are frequently found on mucosal surfaces and in the lower gastrointestinal or urogenital tracts of aquatic and carnivorous vertebrates [61, 62].

The platypus’ reduced stomach, short intestine, and cloacal anatomy may select for microbial taxa distinct from those found in hindgut-fermenting or omnivorous mammals [6, 63]. The cloaca’s integrated digestive and reproductive function likely contributes to the presence of taxa with affinity for mucosal colonisation, some of which may originate from the reproductive tract [43, 61]. The marked divergence between cloacal and faecal microbiota highlights the importance of sampling site and body region when interpreting host–microbe associations.

### Limitations and Future Directions

While this study provides the first broad-scale assessment of the wild platypus cloacal microbiome, several limitations warrant consideration. Sample sizes were uneven across sites, and were relatively small at fire-affected rivers, limiting the ability to disentangle sex-specific or seasonal responses to disturbance or to model finer-scale temporal dynamics. In addition, cloacal swabs capture a composite microbial signal from both digestive and reproductive tracts, complicating direct comparisons with faecal studies [2]. Future work should incorporate longitudinal sampling to examine seasonal or reproductive shifts in microbial composition, and compare juvenile and adult profiles to assess microbiome development. Parallel studies of environmental microbial communities in water and sediment would also clarify the extent to which regional variation reflects external seeding versus host selection. Finally, comparative analyses of wild and captive platypuses, especially during captivity and after release, could inform microbial targets for improved animal care and reintroduction success. Our study provides critical baseline data for this evolutionarily distinct and increasingly threatened species and support the potential utility of microbiome profiling as valuable indicator of host health and environmental change.

## Supplementary Information

Below is the link to the electronic supplementary material.

Supplementary Material 1

## Data Availability

All of the demultiplexed sequence files generated and analysed during this project are available in the NCBI SRA (Short Read Archive) repository under BioProject PRJNA1389828.
