# Supplementary material for: First insights into the Drivers of the Cloacal Microbiome of the Wild Platypus (Ornithorhynchus anatinus)
Source: Microb Ecol. 2026 May 12;89(1):142. doi: 10.1007/s00248-026-02788-1 (PMC13337741; doi:10.1007/s00248-026-02788-1)
Supplement: Supplementary file 1 — Supplementary Material 1 [file 248_2026_2788_MOESM1_ESM.docx]

**Table S1:** Summary of sample locations, sex, age, and breeding season with raw read count, processed read count and whether the sample was removed in rarefication for alpha diversity analyses.

| Sample ID | Raw Read Count | Processed Read Count | River | Region | Sex | Life Stage | Season | Fire | Removed in Rarefying |
| --- | --- | --- | --- | --- | --- | --- | --- | --- | --- |
| 19M230 | 107454 | 78028 | Tidbinbilla | ACT | Female | Adult | Breeding | No | No |
| 19M231 | 157971 | 98964 | Tidbinbilla | ACT | Female | Adult | Breeding | No | No |
| 19M232 | 122582 | 80312 | Tidbinbilla | ACT | Female | Adult | Breeding | No | No |
| 19M233 | 133669 | 99512 | Tidbinbilla | ACT | Female | Adult | Breeding | No | No |
| 19M234 | 5605 | 3435 | Tidbinbilla | ACT | Male | Adult | Breeding | No | Yes |
| 19M235 | 142852 | 92660 | Tidbinbilla | ACT | Male | Adult | Breeding | No | No |
| KV1 | 196700 | 44520 | Kangaroo | Illawarra | Male | Adult | Breeding | No | No |
| KV2 | 99555 | 66723 | Kangaroo | Illawarra | Female | Adult | Breeding | No | No |
| KV3 | 158398 | 127017 | Kangaroo | Illawarra | Female | Adult | Breeding | No | No |
| KV4 | 87780 | 60841 | Kangaroo | Illawarra | Male | Adult | Breeding | No | No |
| KV5 | 151993 | 123490 | Kangaroo | Illawarra | Female | Adult | Breeding | No | No |
| RW2 | 127394 | 94099 | Kangaroo | Illawarra | Female | Adult | Breeding | No | No |
| RW3 | 151059 | 99080 | Kangaroo | Illawarra | Female | Adult | Breeding | No | No |
| RW4 | 188875 | 49398 | Wingecarribee | Illawarra | Male | Sub Adult | Breeding | No | No |
| RW5 | 152282 | 65043 | Wingecarribee | Illawarra | Male | Sub Adult | Breeding | No | No |
| RW6 | 130409 | 41233 | Wingecarribee | Illawarra | Male | Sub Adult | Breeding | No | No |
| KI1 | 4135 | 2095 | Rocky | Kangaroo Island | Male | Juvenile | Non-breeding | Yes16Month | No |
| KI11 | 9759 | 1769 | Rocky | Kangaroo Island | Female | Adult | Breeding | Yes20Month | Yes |
| KI12 | 142901 | 40885 | Rocky | Kangaroo Island | Female | Adult | Breeding | Yes20Month | No |
| KI14 | 212105 | 35127 | Rocky | Kangaroo Island | Male | Juvenile | Breeding | Yes20Month | No |
| KI2 | 169918 | 21144 | Rocky | Kangaroo Island | Female | Juvenile | Non-breeding | Yes16Month | No |
| KI3 | 96132 | 41353 | Rocky | Kangaroo Island | Female | Adult | Non-breeding | Yes16Month | No |
| KI4 | 117072 | 35461 | Rocky | Kangaroo Island | Male | Adult | Non-breeding | Yes16Month | No |
| KI5 | 133109 | 33065 | Rocky | Kangaroo Island | Male | Sub Adult | Non-breeding | Yes16Month | No |
| KI6 | 167195 | 92919 | Rocky | Kangaroo Island | Female | Adult | Non-breeding | Yes16Month | No |
| KI7 | 103584 | 54658 | Rocky | Kangaroo Island | Male | Juvenile | Non-breeding | Yes16Month | No |
| KI8 | 124798 | 73710 | Rocky | Kangaroo Island | Female | Juvenile | Non-breeding | Yes16Month | No |
| KI9 | 22022 | 8728 | Rocky | Kangaroo Island | Male | Adult | Breeding | Yes20Month | Yes |
| D1 | 85428 | 65036 | Dingo | NSW North Coast | Male | Adult | Non-breeding | Yes6month | No |
| D2 | 4484 | 2809 | Dingo | NSW North Coast | Female | Adult | Non-breeding | Yes6month | Yes |
| D3 | 169262 | 108646 | Dingo | NSW North Coast | Male | Adult | Non-breeding | Yes6month | No |
| D4 | 118550 | 78848 | Dingo | NSW North Coast | Female | Adult | Breeding | Yes6month | No |
| D5 | 119038 | 23320 | Dingo | NSW North Coast | Male | Adult | Breeding | Yes6month | No |
| D6 | 116543 | 24688 | Dingo | NSW North Coast | Female | Adult | Non-breeding | Yes18month | No |
| D7 | 3001 | 1439 | Dingo | NSW North Coast | Female | Adult | Non-breeding | Yes18month | Yes |
| D8 | 112865 | 49537 | Dingo | NSW North Coast | Female | Adult | Non-breeding | Yes18month | No |
| T1 | 121516 | 64943 | Thone | NSW North Coast | Male | Adult | Non-breeding | No | No |
| T2 | 128958 | 57528 | Thone | NSW North Coast | Male | Adult | Non-breeding | No | No |
| T3 | 1178 | 418 | Thone | NSW North Coast | Male | Adult | Non-breeding | No | Yes |
| T4 | 165620 | 12711 | Thone | NSW North Coast | Male | Adult | Non-breeding | No | Yes |
| T5 | 2477 | 1185 | Thone | NSW North Coast | Female | Adult | Non-breeding | No | Yes |
| T6 | 190977 | 72830 | Thone | NSW North Coast | Female | Adult | Non-breeding | No | No |
| RW10 | 134011 | 54389 | Bombala | South East NSW | Female | Adult | Breeding | No | No |
| RW11 | 157050 | 35671 | Bombala | South East NSW | Male | Sub Adult | Breeding | No | No |
| RW7 | 864 | 16 | Bombala | South East NSW | Male | Adult | Breeding | No | No |
| RW8 | 2094 | 453 | Bombala | South East NSW | Female | Adult | Breeding | No | Yes |
| RW9 | 97281 | 63598 | Bombala | South East NSW | Female | Adult | Breeding | No | Yes |
| RW12 | 180892 | 81275 | Eucumbene | South East NSW | Female | Adult | Breeding | No | No |
| SX1 | 86127 | 64801 | Snowy | South East NSW | Female | Adult | Breeding | No | No |
| SX10 | 145528 | 56856 | Snowy | South East NSW | Female | Adult | Breeding | No | No |
| SX11 | 138548 | 30011 | Snowy | South East NSW | Male | Adult | Breeding | No | No |
| SX12 | 2947 | 960 | Snowy | South East NSW | Female | Adult | Breeding | No | Yes |
| SX13 | 126811 | 87112 | Snowy | South East NSW | Female | Adult | Breeding | No | No |
| SX14 | 122860 | 63855 | Snowy | South East NSW | Female | Adult | Breeding | No | No |
| SX15 | 158094 | 120204 | Snowy | South East NSW | Female | Adult | Breeding | No | No |
| SX16 | 204958 | 111939 | Snowy | South East NSW | Male | Adult | Breeding | No | No |
| SX17 | 128662 | 81421 | Snowy | South East NSW | Female | Adult | Breeding | No | No |
| SX18 | 156732 | 66134 | Snowy | South East NSW | Female | Adult | Breeding | No | No |
| SX19 | 100458 | 61967 | Snowy | South East NSW | Male | Adult | Breeding | No | No |
| SX2 | 136275 | 74152 | Snowy | South East NSW | Male | Adult | Breeding | No | No |
| SX20 | 148067 | 113017 | Snowy | South East NSW | Female | Adult | Breeding | No | No |
| SX21 | 92888 | 73754 | Snowy | South East NSW | Male | Adult | Breeding | No | No |
| SX22 | 160486 | 39577 | Snowy | South East NSW | Male | Adult | Breeding | No | No |
| SX3 | 82343 | 56130 | Snowy | South East NSW | Female | Adult | Breeding | No | No |
| SX4 | 93788 | 60620 | Snowy | South East NSW | Female | Adult | Breeding | No | No |
| SX5 | 97760 | 71027 | Snowy | South East NSW | Male | Sub Adult | Breeding | No | No |
| SX6 | 135693 | 89477 | Snowy | South East NSW | Male | Sub Adult | Breeding | No | No |
| SX7 | 156494 | 59142 | Snowy | South East NSW | Male | Adult | Breeding | No | No |
| SX8 | 121651 | 79215 | Snowy | South East NSW | Male | Adult | Breeding | No | No |
| SX9 | 134153 | 39515 | Snowy | South East NSW | Female | Adult | Breeding | No | No |


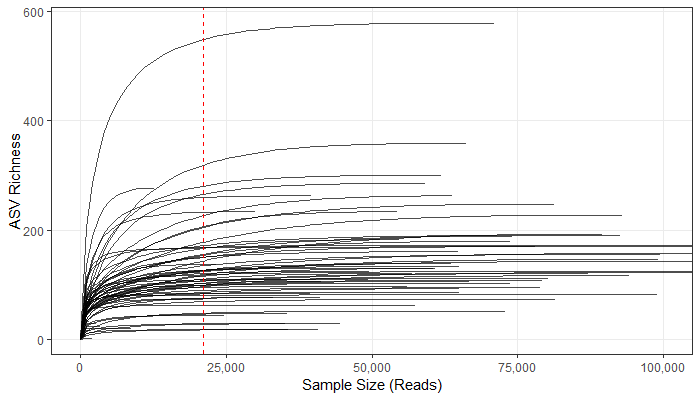


**Figure SX.** Rarefaction curve for all samples retained after quality filtering and decontamination. Each curve represents an individual sample, while the red dashed line indicates the rarefaction threshold of 21,144. Rarefaction curves were visualised using the ‘rarecurve’ function from the R package vegan (v 2.7-3).

**Table S2:** List of identified contaminant ASV’s, Genus and Species which were removed from samples for downstream processing

| ASV Sequence | Genus | Species | Species |
| --- | --- | --- | --- |
| TACGGAGGGTGCGAGCGTTAATCGGAATAACTGGGCGTAAAGGGCACGCAGGCGGTTACTTAAGTGAGATGTGAAATCCCCGAGCTTAACTTGGGAACTGCATTTCATACTGGGTAGCTAGAGTTTTGTAGAGAGGGGTAGAATTCCACGTGTAGCGGTGAAATGCGTAGAGATGTGGAGGAATACCGGAGGCGAAGGCGGCCCCTTGGACAAAGACTGACGCTCAAGTGCGAAAGCGTGGGGAGCAAACAGG | Testudinibacter | NA | NA |
| TACGGAGGGTGCAAGCGTTAATCGGAATTACTGGGCGTAAAGCGCACGCAGGCGGTCTGTCAAGTCGGATGTGAAATCCCCGGGCTCAACCTGGGAACTGCATTCGAAACTGGCAGGCTAGAGTCTTGTAGAGGGGGGTAGAATTCCAGGTGTAGCGGTGAAATGCGTAGAGATCTGGAGGAATACCGGTGGCGAAGGCGGCCCCCTGGACAAAGACTGACGCTCAGGTGCGAAAGCGTGGGGAGCAAACAGG | Klebsiella | NA | NA |
| TACGTAGGTGGCAAGCGTTGTCCGGATTTATTGGGCGTAAAGCGAGAGCAGGCGGTCTCTTAAGTCTGATGTGAAAGCCCACGGCTCAACCGTGGAACTGCATTGGAAACTGGGAGACTTGAGTGCAGAAGAGGAGAGTGGAATTCCATGTGTAGCGGTAAAATGCGGAGATATATGGAGGAACACCAGTGGCGAAGGCGACTCTCTGGTCTGTAACTGACGCTGAGTCTCGAAAGCGTGGGGAGCAAACAGG | NA | NA | NA |
| TACGTAGGGGGCTAGCGTTATCCGGAATTACTGGGCGTAAAGGGTGCGTAGGTGGTTTCTTAAGCCAGAAGTGAAAGGCTACGGCTCAACCGTAGTAAGCTTTTGGAACTGGGAAACTTGAGTGCAGGAGAGGAGAGTAGAATTCCTAGTGTAGCGGTGAAATGCGTAGATATTAGGAGGAATACCAGTAGCGAAGGCGGCTCTCTGGACTGTAACTGACACTGAGGCACGAAAGCGTGGGGAGCAAACAGG | Paraclostridium | NA | NA |
| TACGGAGGGTGCGAGCGTTAATCGGAATAACTGGGCGTAAAGGGCACGCAGGCGGCCACTTAAGTGAGATGTGAAATCCCCGAGCTTAACTTGGGAACTGCATTTCATACTGGGTGGCTAGAGTCTTGTAGAGAGGGGTAGAATTCCACGTGTAGCGGTGAAATGCGTAGAGATGTGGAGGAATACCGGAGGCGAAGGCGGCCCCTTGGACAAAGACTGACGCTCATGTGCGAAAGCGTGGGGAGCAAACAGG | Testudinibacter | NA | NA |
| TACGTAGGGCGCAAGCGTTATCCGGAATTATTGGGCGTAAAGAGCTTGTAGGCGGTTTGTCGCGTCTGCTGTGAAAGACCGGGGCTCAACTCCGGTTCTGCAGTGGGTACGGGCAGACTAGAGTGATGTAGGGGAGACTGGAATTCCTGGTGTAGCGGTGAAATGCGCAGATATCAGGAGGAACACCGATGGCGAAGGCAGGTCTCTGGGCATTAACTGACGCTGAGAAGCGAAAGCATGGGGAGCGAACAGG | Arthrobacter | NA | NA |
| TACGTAGGTGGCAAGCGTTGTCCGGATTTACTGGGCGTAAAGGGAGCGTAGGCGGACTTTTAAGTGAGATGTGAAATACCCGAGCTTAACTTGGGTGCTGCATTTCAAACTGGAAGTCTAGAGTGCAGGAGAGGAGAATGGAATTCCTAGTGTAGCGGTGAAATGCGTAGAGATTAGGAAGAACACCAGTGGCGAAGGCGATTCTCTGGACTGTAACTGACGCTGAGGCTCGAAAGCGTGGGGAGCAAACAGG | Clostridium | NA | NA |
| TACGAAGGGTGCAAGCGTTAATCGGAATTACTGGGCGTAAAGCGCGCGTAGGTGGTTCGTTAAGTTGGATGTGAAAGCCCCGGGCTCAACCTGGGAACTGCATCCAAAACTGGCGAGCTAGAGTATGGCAGAGGGTGGTGGAATTTCCTGTGTAGCGGTGAAATGCGTAGATATAGGAAGGAACACCAGTGGCGAAGGCGACCACCTGGGCTAATACTGACACTGAGGTGCGAAAGCGTGGGGAGCAAACAGG | Pseudomonas | stutzeri | NA |
| TACGTAGGGGGCAAGCGTTATCCGGAATTACTGGGTGTAAAGGGTGCGTAGGTGGCGATTTAAGTCAGATGTGAAAACTCAGGGCTCAACCTTGAGACTGCATCTGAAACTGAGTTGCTAGAGTGCAGGAGAGGAAAGCGGAATTCCGAGTGTAGCGGTGAAATGCGTAGAGATTCGGAGGAACACCAGTAGCGAAGGCGGCTTTCTGGACTGTAACTGACACTGAGGCACGAAAGCGTGGGGAGCGAACAGG | Epulopiscium | NA | NA |
| TACGAAGGGTGCAAGCGTTAATCGGAATTACTGGGCGTAAAGCGCGCGTAGGTGGTTCAGCAAGTTGGATGTGAAATCCCCGGGCTCAACCTGGGAACTGCATCCAAAACTACTGAGCTAGAGTACGGTAGAGGGTGGTGGAATTTCCTGTGTAGCGGTGAAATGCGTAGATATAGGAAGGAACACCAGTGGCGAAGGCGACCACCTGGACTGATACTGACACTGAGGTGCGAAAGCGTGGGGAGCAAACAGG | Pseudomonas | aeruginosa | NA |
| TACAGAGGGTGCGAGCGTTAATCGGATTTACTGGGCGTAAAGCGTGCGTAGGCGGCTTTTTAAGTCGGATGTGAAATCCCTGAGCTTAACTTAGGAATTGCATTCGATACTGGGAAGCTAGAGTATGGGAGAGGATGGTAGAATTCCAGGTGTAGCGGTGAAATGCGTAGAGATCTGGAGGAATACCGATGGCGAAGGCAGCCATCTGGCCTAATACTGACGCTGAGGTACGAAAGCATGGGGAGCAAACAGG | Acinetobacter | johnsonii | NA |
| TACAGAGGGTGCAAGCGTTAATCGGAATTACTGGGCGTAAAGCGCGCGTAGGTGGTTTGTTAAGTTGGATGTGAAAGCCCCGGGCTCAACCTGGGAACTGCATCCAAAACTGGCAAGCTAGAGTACGGTAGAGGGTGGTGGAATTTCCTGTGTAGCGGTGAAATGCGTAGATATAGGAAGGAACACCAGTGGCGAAGGCGACCACCTGGACTGATACTGACACTGAGGTGCGAAAGCGTGGGGAGCAAACAGG | Pseudomonas | NA | NA |
| TACAGAGGGTGCAAGCGTTAATCGGAATTACTGGGCGTAAAGCGCGCGTAGGTGGTTTGTTAAGTTGGATGTGAAATCCCCGGGCTCAACCTGGGAACTGCATTCAAAACTGACTGACTAGAGTATGGTAGAGGGTGGTGGAATTTCCTGTGTAGCGGTGAAATGCGTAGATATAGGAAGGAACACCAGTGGCGAAGGCGACCACCTGGACTAATACTGACACTGAGGTGCGAAAGCGTGGGGAGCAAACAGG | Pseudomonas | antarctica | NA |
| TACGGAGGGTGCAAGCGTTAATCGGAATTACTGGGCGTAAAGCGCACGCAGGCGGTTTGTTAAGTCAGATGTGAAATCCCCGGGCTCAACCTGGGAACTGCATCTGATACTGGCAAGCTTGAGTCTCGTAGAGGGGGGTAGAATTCCAGGTGTAGCGGTGAAATGCGTAGAGATCTGGAGGAATACCGGTGGCGAAGGCGGCCCCCTGGACGAAGACTGACGCTCAGGTGCGAAAGCGTGGGGAGCAAACAGG | Escherichia-Shigella | NA | NA |
| TACGGAGGGCGCGAGCGTTACCCGGATTCACTGGGCGTAAAGGGCGTGTAGGCGGCCTGGGGCGTCCCATGTGAAAGACCACGGCTCAACCGTGGGGGAGCGTGGGATACGCTCAGGCTAGACGGTGGGAGAGGGTGGTGGAATTCCCGGAGTAGCGGTGAAATGCGCAGATACCGGGAGGAACGCCGATGGCGAAGGCAGCCACCTGGTCCACCCGTGACGCTGAGGCGCGAAAGCGTGGGGAGCAAACCGG | Thermus | thermophilus | NA |
| TACGTAGGGGGCGAGCGTTGTCCGGAATTATTGGGCGTAAAGCGCGCGCAGGCGGTCCCTTAAGTCTGATGTGAAAGCCCACGGCTCAACCGTGGAGGGTCATTGGAAACTGGGGGACTTGAGTGCAGAAGAGGAGAGCGGAATTCCACGTGTAGCGGTGAAATGCGTAGAGATGTGGAGGAACACCAGTGGCGAAGGCGGCTCTCTGGTCTGTAACTGACGCTGAGGCGCGAAAGCGTGGGGAGCAAACAGG | Parageobacillus | toebii | NA |
| TACGTAGGTGGCAAGCGTTATCCGGAATTATTGGGCGTAAAGCGCGCGTAGGCGGTTTTTTAAGTCTGATGTGAAAGCCCACGGCTCAACCGTGGAGGGTCATTGGAAACTGGAAAACTTGAGTGCAGAAGAGGAAAGTGGAATTCCATGTGTAGCGGTGAAATGCGCAGAGATATGGAGGAACACCAGTGGCGAAGGCGACTTTCTGGTCTGTAACTGACGCTGATGTGCGAAAGCGTGGGGATCAAACAGG | Staphylococcus | aureus | NA |
| TACGGAGGGTGCAAGCGTTAATCGGAATTACTGGGCGTAAAGCGCACGCAGGCGGCTTTTTAAGTCGGATGTGAAAGCCCCGGGCTCAACCTGGGAATTGCATCTGATACTGGGAAGCTAGAGTATGTGAGAGGGGGGTAGAATTCCAAGTGTAGCGGTGAAATGCGTAGAGATTTGGAGGAATACCAGTGGCGAAGGCGGCCCCCTGGCACAATACTGACGCTCAGGTGCGAAAGCGTGGGGAGCAAACAGG | Alishewanella | NA | NA |
| TACGGAGGGAGCTAGCGTTGTTCGGAATTACTGGGCGTAAAGCGCACGTAGGCGGCGATTTAAGTCAGAGGTGAAAGCCCGGGGCTCAACCCCGGAACTGCCTTTGAGACTGGATTGCTTGAATCCTGGAGAGGTGAGTGGAATTCCGAGTGTAGAGGTGAAATTCGTAGATATTCGGAAGAACACCAGTGGCGAAGGCGGCTCACTGGACAGGTATTGACGCTGAGGTGCGAAAGCGTGGGGAGCAAACAGG | Sphingobium | amiense | NA |
| TACGTAGGGTGCGAGCGTTAATCGGAATTACTGGGCGTAAAGCGTGCGCAGGCGGCTTTGCAAGACAGAGGTGAAATCCCCGGGCTCAACCTGGGAACTGCCTTTGTGACTGCAAGGCTAGAGTACGGCAGAGGGGGATGGAATTCCGCGTGTAGCAGTGAAATGCGTAGATATGCGGAGGAACACCAATGGCGAAGGCAATCCCCTGGGCCTGTACTGACGCTCATGCACGAAAGCGTGGGGAGCAAACAGG | Aquabacterium | NA | parvum |
| TACGGAGGGTCCGAGCGTTAATCGGAATTACTGGGCGTAAAGCGTGCGCAGGCGGTTTGTTAAGCGAGATGTGAAAGCCCTGGGCTCAACCTAGGAATAGCATTTCGAACTGGCGAACTAGAGTCTTGTAGAGGGGGGTAGAATTCCAGGTGTAGCGGTGAAATGCGTAGAGATCTGGAGGAATACCGGTGGCGAAGGCGGCCCCCTGGACAAAGACTGACGCTCATGCACGAAAGCGTGGGGAGCAAACAGG | Shewanella | putrefaciens | NA |
| TACGTAGGGTGCGAGCGTTGTCCGGAATTACTGGGCGTAAAGAGCTCGTAGGCGGTTTGTCACGTCGTCTGTGAAATCCTAGGGCTTAACCCTGGACGTGCAGGCGATACGGGCTGACTTGAGTACTACAGGGGAGACTGGAATTTCTGGTGTAGCGGTGGAATGCACAGATATCAGGAAGAACACCGATGGCGAAGGCAGGTCTCTGGGTAGTAACTGACGCTGAGGAGCGAAAGCATGGGTAGCGAACAGG | Lawsonella | clevelandensis | clevelandensis |
| TACGTAGGGTGCAAGCGTTAATCGGAATTACTGGGCGTAAAGCGTGCGCAGGCGGTCCGCTAAGACAGATGTGAAATCCCCGGGCTTAACCTGGGAACTGCATTTGTGACTGGCGGGCTAGAGTATGGCAGAGGGGGGTAGAATTCCACGTGTAGCAGTGAAATGCGTAGAGATGTGGAGGAATACCGATGGCGAAGGCAGCCCCCTGGGCCAATACTGACGCTCATGCACGAAAGCGTGGGGAGCAAACAGG | Burkholderia-Caballeronia-Paraburkholderia | NA | NA |
| TACGGAGGGTGCGAGCGTTACCCGGATTTACTGGGCGTAAAGGGCGTGTAGGCGGTTTTCTAAGTCCGGGGCTAAAGACCAGGGCTCAACCCTGGGTTTGCCTTGGATACTGGAAAGCTCGACGGCTGGAGGGGGCAGCGGAATTTCCGGAGTAGCGGTGAAATGCGCAGATACCGGAAGGAACGCCAATAGCGAAGGCAGCTGCCTGGACAGTACGTGACGCTGAGGCGCGAAAGCGTGGGGAGCAAACCGG | Allomeiothermus | NA | NA |
| TACGTAGGGTGCAAGCGTTAATCGGAATTACTGGGCGTAAAGCGTGCGCAGGCGGTTGTGTAAGTCAGATGTGAAATCCCCGGGCTCAACCTGGGAATTGCATTTGAGACTGCACGGCTAGAGTGTGTCAGAGGGGGGTAGAATTCCACGTGTAGCAGTGAAATGCGTAGATATGTGGAGGAATACCGATGGCGAAGGCAGCCCCCTGGGATAACACTGACGCTCATGCACGAAAGCGTGGGGAGCAAACAGG | Herbaspirillum | huttiense | NA |
| TACGTAGGTGGCAAGCGTTATCCGGAATTATTGGGCGTAAAGCGCGCGTAGGCGGTTTCTTAAGTCTGATGTGAAAGCCCACGGCTCAACCGTGGAGGGTCATTGGAAACTGGGAAACTTGAGTGCAGAAGAGGAAAGTGGAATTCCATGTGTAGCGGTGAAATGCGCAGAGATATGGAGGAACACCAGTGGCGAAGGCGACTTTCTGGTCTGTAACTGACGCTGATGTGCGAAAGCGTGGGGATCAAACAGG | Staphylococcus | saprophyticus | NA |
| TACGAAGGGGGCTAGCGTTGTTCGGAATTACTGGGCGTAAAGCGCACGTAGGCGGATATTTAAGTCAGGGGTGAAATCCCAGAGCTCAACTCTGGAACTGCCTTTGATACTGGGTATCTTGAGTATGGAAGAGGTAAGTGGAATTCCGAGTGTAGAGGTGAAATTCGTAGATATTCGGAGGAACACCAGTGGCGAAGGCGGCTTACTGGTCCATTACTGACGCTGAGGTGCGAAAGCGTGGGGAGCAAACAGG | Agrobacterium | NA | NA |
| TACGTAGGTGGCAAGCGTTGTCCGGATTTATTGGGCGTAAAGGGAGCGCAGGCGGTTCGTTAAGTCTGATGTGAAAGCCCTCGGCTCAACCGAGGAGGGTCATTGGAAACTGGCGAACTTGAGTGCAGAAGAGGAGAGTGGAATTCCATGTGTAGCGGTGAAATGCGTAGATATATGGAGGAACACCGGTGGCGAAGGCGACTCTCTGGTCTGTAACTGACGCTGAGGCTCGAAAGCGTGGGTAGCAAACAGG | Tetragenococcus | NA | osmophilus |
| TACAGAGGGTGCAAGCGTTAATCGGATTTACTGGGCGTAAAGCGCGCGTAGGTGGCCAATTAAGTCAAATGTGAAATCCCCGAGCTTAACTTGGGAATTGCATTCGATACTGGTTGGCTAGAGTATGGGAGAGGATGGTAGAATTCCAGGTGTAGCGGTGAAATGCGTAGAGATCTGGAGGAATACCGATGGCGAAGGCAGCCATCTGGCCTAATACTGACACTGAGGTGCGAAAGCATGGGGAGCAAACAGG | Acinetobacter | lwoffii | lwoffii |
| TACGTAGGGCGCAAGCGTTGTCCGGAATTATTGGGCGTAAAGAGCTTGTAGGTGGCTTGTCGCGTCTGCCGTGAAAACCCGAGGCTCAACCTCGGACGTGCGGTGGGTACGGGCAGGCTAGAGTGTGGTAGGGGAGACTGGAACTCCTGGTGTAGCGGTGAAATGCGCAGATATCAGGAAGAACACCGATGGCGAAGGCAGGTCTCTGGGCCATTACTGACACTGAGAAGCGAAAGCATGGGGAGCGAACAGG | Brachybacterium | NA | NA |
| TACGTAGGGTGCAAGCGTTAATCGGAATTACTGGGCGTAAAGCGTGCGCAGGCGGTTTTGTAAGTCTGTCGTGAAAGCCCCGGGCTCAACCTGGGAATTGCGATGGAGACTGCAAGGCTTGAATCTGGCAGAGGGGGGTAGAATTCCACGTGTAGCAGTGAAATGCGTAGAGATGTGGAGGAACACCGATGGCGAAGGCAGCCCCCTGGGTCAAGATTGACGCTCATGCACGAAAGCGTGGGGAGCAAACAGG | Massilia | NA | NA |
| TACAGAGGTCTCAAGCGTTGTTCGGAATCACTGGGCGTAAAGAGTACGTAGGCTGTTTTCTAAGTCAGGTGTGAAAGGCAGGGGCTCAACCCCTGGACTGCACATGATACTGGGAGACTGGAGTAATGGAGGGGGAACCGGAATTCTCGGTGTAGCAGTGAAATGCGTAGATATCGAGAGGAACACTCGTGGCGAAGGCGGGTTCCTGGACATTAACTGACGCTGAGGTACGAAGGCCAGGGTAGCGAAAGGG | Akkermansia | NA | NA |
| TACGTAGGGTGCAAGCGTTAATCGGAATTACTGGGCGTAAAGCGTGCGCAGGCGGTTCGCTAAGACAGATGTGAAATCCCCGGGCTTAACCTGGGAACTGCATTTGTGACTGGCGGGCTAGAGTATGGCAGAGGGGGGTAGAATTCCACGTGTAGCAGTGAAATGCGTAGAGATGTGGAGGAATACCGATGGCGAAGGCAGCCCCCTGGGCCAATACTGACGCTCATGCACGAAAGCGTGGGGAGCAAACAGG | Burkholderia-Caballeronia-Paraburkholderia | NA | NA |
| TACGTAGGTCCCGAGCGTTGTCCGGATTTATTGGGCGTAAAGCGAGCGCAGGCGGTTAGATAAGTCTGAAGTTAAAGGCTGTGGCTTAACCATAGTACGCTTTGGAAACTGTTTAACTTGAGTGCAAGAGGGGAGAGTGGAATTCCATGTGTAGCGGTGAAATGCGTAGATATATGGAGGAACACCGGTGGCGAAAGCGGCTCTCTGGCTTGTAACTGACGCTGAGGCTCGAAAGCGTGGGGAGCAAACAGG | Streptococcus | oralis | NA |
| TACGGAGGGTGCGAGCGTTAATCGGAATAACTGGGCGTAAAGGGCACGCAGGCGGTGACTTAAGTGAGGTGTGAAAGCCCCGGGCTTAACCTGGGAATTGCATTTCATACTGGGTCGCTAGAGTACTTTAGGGAGGGGTAGAATTCCACGTGTAGCGGTGAAATGCGTAGAGATGTGGAGGAATACCGAAGGCGAAGGCAGCCCCTTGGGAATGTACTGACGCTCATGTGCGAAAGCGTGGGGAGCAAACAGG | Haemophilus | NA | NA |
| TACAGAGGGTGCGAGCGTTAATCGGATTTACTGGGCGTAAAGCGTGCGTAGGCGGCTTCTTAAGTCGGATGTGAAATCCCTGAGCTTAACTTAGGAATTGCATTCGATACTGGGAAGCTAGAGTATGGGAGAGGATGGTAGAATTCCAGGTGTAGCGGTGAAATGCGTAGAGATCTGGAGGAATACCGATGGCGAAGGCAGCCATCTGGCCTAATACTGACGCTGAGGTACGAAAGCATGGGGAGCAAACAGG | Acinetobacter | johnsonii | NA |
| TACGGAGGGTGCAAGCGTTGTCCGGATTTATTGGGTTTAAAGGGTGCGTAGGCGGCGTAATAAGTCCGTGGTGAAAGCCAACAGCTCAACTGTTGAACTGCCATGGATACTGTTAGGCTTGAGTACAGACGAGGTAGGCGGAATGGATCGTGTAGCGGTGAAATGCATAGATACGATCCAGAACTCCGATTGCGAAGGCAGCTTACTAGGCTGTAACTGACGCTGAGGCACGAAAGCGTGGGGAGCGAACAGG | Adhaeribacter | NA | NA |
| TACGTAGGGTGCAAGCGTTAATCGGAATTACTGGGCGTAAAGCGTGCGCAGGCGGTTATATAAGACAGATGTGAAATCCCCGGGCTCAACCTGGGAACTGCATTTGTGACTGTATAGCTAGAGTACGGCAGAGGGGGATGGAATTCCGCGTGTAGCAGTGAAATGCGTAGATATGCGGAGGAACACCGATGGCGAAGGCAATCCCCTGGGCCTGTACTGACGCTCATGCACGAAAGCGTGGGGAGCAAACAGG | Acidovorax | carolinensis | NA |
| TACGGAGGGTGCGAGCGTTAATCGGAATTACTGGGCGTAAAGCGCGCGTAGGCGGCGTGATAAGCCGGTTGTGAAAGCCCCGGGCTCAACCTGGGAACGGCATCCGGAACTGTCAGGCTAGAGTGCAGGAGAGGAAGGTAGAATTCCCGGTGTAGCGGTGAAATGCGTAGAGATCGGGAGGAATACCAGTGGCGAAGGCGGCCTTCTGGACTGACACTGACGCTGAGGTGCGAAAGCGTGGGTAGCAAACAGG | Halomonas | ventosae | NA |
| TACGTAGGTGGCGAGCGTTGTCCGGATTTATTGGGCGTAAAGGGAGTGTAGGCGGTCTTTTAAGTCTGATGTGAAAGCCCACGGCTCAACCGTGGAGGGTCATTGGAAACTGGGAGACTTGAGTGCAGAAGAGGAGAGCGGAATTCCATGTGTAGCGGTGAAATGCGTAGATATATGGAGGAACACCAGTGGCGAAGGCGGCTCTCTGGTCTGTAACTGACGCTGAGGCTCGAAAGCGTGGGGAGCAAACAGG | Abiotrophia | NA | defectiva |
| TACGTAGGGTGCGAGCGTTGTCCGGAATTACTGGGCGTAAAGGGCTCGTAGGTGGTTTGTCGCGTCGTCTGTGAAATTCCGGGGCTTAACTCCGGGCGTGCAGGCGATACGGGCATAACTTGAGTACTGTAGGGGTAACTGGAATTCCTGGTGTAGCGGTGAAATGCGCAGATATCAGGAGGAACACCGATGGCGAAGGCAGGTTACTGGGCAGTTACTGACGCTGAGGAGCGAAAGCATGGGTAGCGAACAGG | Corynebacterium | tuberculostearicum | NA |
| TACGAAGGGGGCTAGCGTTGCTCGGAATCACTGGGCGTAAAGGGTGCGTAGGCGGGTTTTTAAGTCAGAGGTGAAATCCTGGAGCTCAACTCCAGAACTGCCTTTGATACTGGGAATCTTGAGTATGGAAGAGGTGAGTGGAACTGCGAGTGTAGAGGTGAAATTCGTAGATATTCGCAAGAACACCAGTGGCGAAGGCGGCTCACTGGTCCATAACTGACGCTGAGGCACGAAAGCGTGGGGAGCAAACAGG | Rhodopseudomonas | NA | NA |
| TACGTAGGGCGCGAGCGTTGTCCGGAATTATTGGGCGTAAAGAGCTCGTAGGCGGCTGGTCGCGTCTGTCGTGAAATCCTCTGGCTTAACTGGGGGCTTGCGGTGGGTACGGGCCGGCTTGAGTGCGGTAGGGGAGACTGGAACTCCTGGTGTAGCGGTGGAATGCGCAGATATCAGGAAGAACACCGGTGGCGAAGGCGGGTCTCTGGGCCGTTACTGACGCTGAGGAGCGAAAGCGTGGGGAGCGAACAGG | Actinomyces | naeslundii | NA |
| TACGGAGGGTGCAAGCGTTACCCGGAATCACTGGGCGTAAAGGGCGTGTAGGCGGAAGGTTAAGTCCGACTTTAAAGACCGGGGCTCAACCCCGGGCCTGGGTTGGAGACTGGCTTTCTGGACCTCTGGAGAGGCAACTGGAATTCCTGGTGTAGCGGTGGAATGCGTAGATACCAGGAGGAACACCGATGGCGAAGGCAGGTTGCTGGACAGAAGGTGACGCTGAGGCGCGAAAGTGTGGGGAGCGAACCGG | Deinococcus | geothermalis | geothermalis |
| TACGAAGGGGGCTAGCGTTGCTCGGAATTACTGGGCGTAAAGGGAGCGTAGGCGGACATTTAAGTCAGGGGTGAAATCCCGGGGCTCAACCTCGGAATTGCCTTTGATACTGGGTGTCTTGAGTATGAGAGAGGTGTGTGGAACTCCGAGTGTAGAGGTGAAATTCGTAGATATTCGGAAGAACACCAGTGGCGAAGGCGACACACTGGCTCATTACTGACGCTGAGGCTCGAAAGCGTGGGGAGCAAACAGG | Brevundimonas | vesicularis | NA |
| TACGTAGGGTGCGAGCGTTGTCCGGAATTACTGGGCGTAAAGAGCTCGTAGGTGGTCTGTCGCGTCATTTGTGAAAGCCCGGGGCTTAACTCCGGGTTGGCAGGTGATACGGGCATGACTGGAGTACTGTAGGGGAGACTGGAATTCCTGGTGTAGCGGTGAAATGCGCAGATATCAGGAGGAACACCGGTGGCGAAGGCGGGTCTCTGGGCAGTAACTGACGCTGAGGAGCGAAAGCATGGGTAGCGAACAGG | Corynebacterium | kroppenstedtii | kroppenstedtii |
| TACGTAGGTGGCAAGCGTTGTCCGGATTTACTGGGCGTAAAGGGAGCGTAGGTGGATATTTAAGTGGGATGTGAAATACTCGGGCTTAACCTGGGTGCTGCATTCCAAACTGGATATCTAGAGTGCAGGAGAGGAAAGGAGAATTCCTAGTGTAGCGGTGAAATGCGTAGAGATTAGGAAGAATACCAGTGGCGAAGGCGCCTTTCTGGACTGTAACTGACACTGAGGCTCGAAAGCGTGGGGAGCAAACAGG | Clostridium | butyricum | butyricum |
| TACAGAGGGTGCGAGCGTTAATCGGATTTACTGGGCGTAAAGCGTGCGTAGGCGGCTTATTAAGTCGGATGTGAAATCCCCGAGCTTAACTTGGGAATTGCATTCGATACTGGTGAGCTAGAGTATGGGAGAGGATGGTAGAATTCCAGGTGTAGCGGTGAAATGCGTAGAGATCTGGAGGAATACCGATGGCGAAGGCAGCCATCTGGCCTAATACTGACGCTGAGGTACGAAAGCATGGGGAGCAAACAGG | Acinetobacter | baumannii | NA |
| TACGTAGGGGGCGAGCGTTGTCCGGAATTATTGGGCGTAAAGCGCGCGCAGGCGGTCCCTTAAGTCTGATGTGAAAGCCCACGGCTCAACCGTGGAGGGGCATTGGAAACTGGGGGACTTGAGTGCAGAAGAGGAGAGCGGAATTCCACGTGTAGCGGTGAAATGCGTAGAGATGTGGAGGAACACCAGTGGCGAAGGCGGCTCTCTGGTCTGTAACTGACGCTGAGGCGCGAAAGCGTGGGGAGCAAACAGG | Parageobacillus | NA | NA |
| TACGAAGGGGGCTAGCGTTGTTCGGAATTACTGGGCGTAAAGCGCACGTAGGCGGATATTTAAGTCAGGGGTGAAATCCCAGAGCTCAACTCTGGAACTGCCTTTGATACTGGGTATCTTGAGTATGGAAGAGGTGAGTGGAATTCCGAGTGTAGAGGTGAAATTCGTAGATATTCGGAGGAACACCAGTGGCGAAGGCGGCTCACTGGTCCATAACTGACGCTGAGGTGCGAAAGCGTGGGGAGCAAACAGG | NA | NA | NA |
| TACGTAGGGGGCAAGCGTTGTCCGGATTTATTGGGCGTAAAGAGCGTGTAGGCGGCCATGTAGGTCCGTTGTGAAAACTCGAGGCTCAACCTCGAGACGCCGATGGAAACCATGTGGCTAGAGTCCGGAAGAGGAGAGTGGAATTCCTGGTGTAGCGGTGAAATGCGCAGATATCAGGAAGAACACCCGTGGCTAAGGCGGCTCTCTAGTACGGTACTGACGCTGAGACGCGAAAGCGTGGGGAGCGAACAGG | NA | NA | NA |
| TACGAAGGGGGCTAGCGTTGCTCGGAATTACTGGGCGTAAAGGGCGCGTAGGCGGACAGTTTAGTCAGAGGTGAAAGCCCAGGGCTCAACCTTGGAATTGCCTTTGATACTGGCTGTCTTGAGTTCGGGAGAGGTGAGTGGAATGCCGAGTGTAGAGGTGAAATTCGTAGATATTCGGCGGAACACCAGTGGCGAAGGCGACTCACTGGCCCGATACTGACGCTGAGGCGCGAAAGCGTGGGGAGCAAACAGG | Phenylobacterium | NA | mobile |
| TACGTAGGGTGCGAGCGTTGTCCGGAATTACTGGGCGTAAAGAGCTCGTAGGTGGTTTGTCGCGTCGTCTGTGAAATTCCGGGGCTTAACTCCGGGCGTGCAGGCGATACGGGCATAACTTGAGTACTGTAGGGGAGACTGGAATTCCTGGTGTAGCGGTGAAATGCGCAGATATCAGGAGGAACACCGGTGGCGAAGGCGGGTCTCTGGGCAGTAACTGACGCTGAGGAGCGAAAGCATGGGGAGCAAACAGG | Corynebacterium | NA | NA |

**
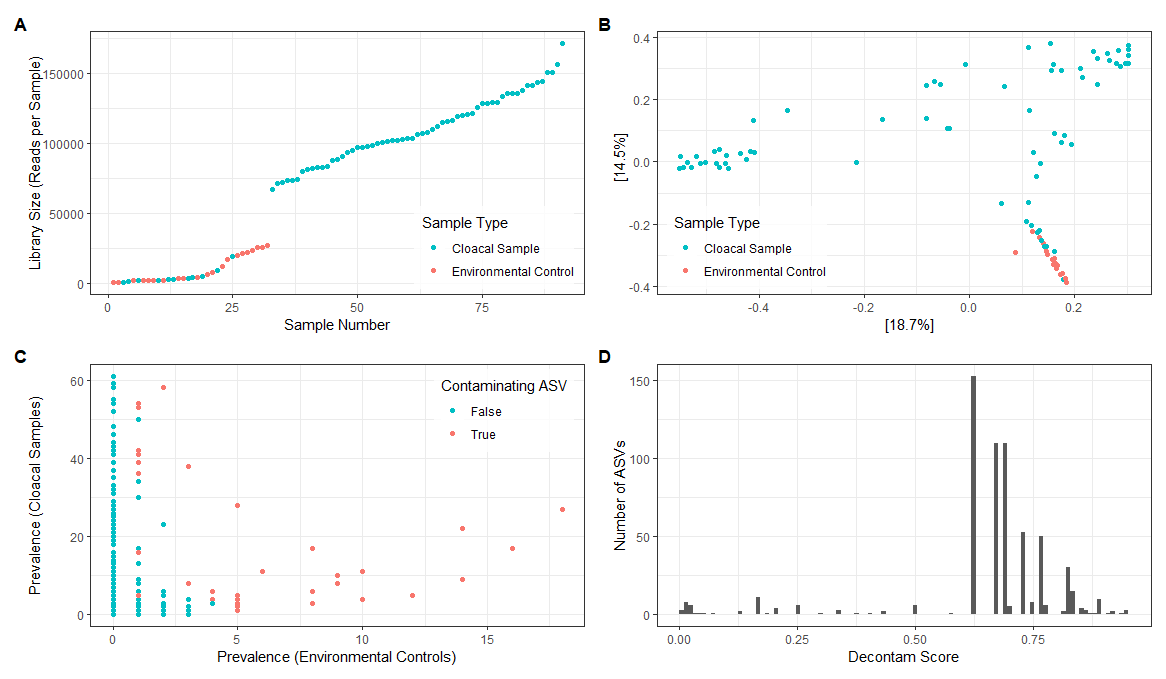
**

**Figure S2.**Summary plots of the *decontam* pipeline, where (A) demonstrates the library sizes of cloacal samples and environmental controls prior to decontamination, (B) Principal Coordinates Analysis (PCoA) of Bray-Curtis dissimilarity between cloacal samples and environmental controls coloured by sample type, (C) the prevalence of ASVs identified as contaminants in cloacal samples and environmental controls at a probability threshold of 0.5, and (D) the distribution of ASVs across *decontam* scores (*p*-values).

**Table S3:** List of taxa identified as core members of the microbiome present in 70% of samples within each region at 0.001 abundance minimum.

| **Location** | **Phym** | **Family** | **Genus** | **Mean_abundance** | **SD_abundance** | **SE_abundance** | **Total_abundance** | **N** |
| --- | --- | --- | --- | --- | --- | --- | --- | --- |
| ACT | Bacillota | Streptococcaceae | Lactococcus | 0.011565134 | 0.046074791 | 0.005027172 | 0.97147124 | 84 |
| ACT | Bacillota | Enterococcaceae | Enterococcus | 0.005352254 | 0.027322084 | 0.002187517 | 0.834951629 | 156 |
| ACT | Bacillota | Peptostreptococcaceae | Paraclostridium | 0.004078303 | 0.033839727 | 0.002709347 | 0.636215288 | 156 |
| ACT | Bacillota | Peptostreptococcaceae | Terrisporobacter | 0.001632701 | 0.010323469 | 0.000692866 | 0.362459665 | 222 |
| ACT | Bacillota | Lachnospiraceae | Epulopiscium | 0.000531674 | 0.003091662 | 0.000173372 | 0.169072377 | 318 |
| ACT | Bacillota | Peptostreptococcaceae | Romboutsia | 0.000450633 | 0.005659029 | 0.000265008 | 0.205488529 | 456 |
| ACT | Bacillota | Clostridiaceae | Clostridium | 0.000445356 | 0.004141799 | 9.3363356e-05 | 0.876460458 | 1968 |
| ACT | Bacteroidota | Bacteroidaceae | Bacteroides | 0.002519422 | 0.015024912 | 0.00089472 | 0.710476897 | 282 |
| ACT | Fusobacteriota | Fusobacteriaceae | Fusobacterium | 0.002642612 | 0.016503066 | 0.001052197 | 0.650082656 | 246 |
| ACT | Pseudomonadota | Enterobacteriaceae | Citrobacter | 0.019444042 | 0.08526629 | 0.015567423 | 0.583321263 | 30 |
| Illawarra | Actinomycetota | Dermacoccaceae | Dermacoccus | 0.037847103 | 0.134554562 | 0.017370919 | 2.270826175 | 60 |
| Illawarra | Pseudomonadota | Enterobacteriaceae | Plesiomonas | 0.048034878 | 0.191027619 | 0.018213769 | 5.283836627 | 110 |
| Illawarra | Pseudomonadota | Neisseriaceae | Alysiella | 0.034933389 | 0.135450886 | 0.016189477 | 2.445337197 | 70 |
| Kangaroo Island | Campylobacterota | Campylobacteraceae | Campylobacter | 0.05 | 0.217836434 | 0.014061281 | 9 | 240 |
| NSW North Coast | Actinomycetota | Dermacoccaceae | Dermacoccus | 0.013658916 | 0.060243121 | 0.006573063 | 1.065395468 | 84 |
| NSW North Coast | Bacillota | Peptostreptococcaceae | Testudinibacter | 0.00551813 | 0.045088083 | 0.002066609 | 2.439013413 | 476 |
| NSW North Coast | Bacillota | Peptostreptococcaceae | Romboutsia | 0.003123998 | 0.042304082 | 0.001296915 | 3.086510043 | 1064 |
| NSW North Coast | Campylobacterota | Campylobacteraceae | Campylobacter | 0.012806658 | 0.091599452 | 0.005474114 | 3.329731118 | 280 |
| NSW North Coast | Pseudomonadota | Neisseriaceae | Alysiella | 0.033839011 | 0.117458987 | 0.01186515 | 3.079349958 | 98 |
| South East NSW | Bacillota | Peptostreptococcaceae | Romboutsia | 0.003951098 | 0.048288033 | 0.001046776 | 8.107652752 | 2128 |
| South East NSW | Campylobacterota | Campylobacteraceae | Campylobacter | 0.019017532 | 0.120899763 | 0.005108947 | 10.26946747 | 560 |
| South East NSW | Fusobacteriota | Fusobacteriaceae | Fusobacterium | 0.005782464 | 0.050310604 | 0.001484871 | 6.401187203 | 1148 |
| South East NSW | Pseudomonadota | Aeromonadaceae | Aeromonas | 0.008228491 | 0.05522931 | 0.003300583 | 2.221692576 | 280 |
